# DNA methylation of hematopoietic stem/progenitor cells from donor peripheral blood to patient bone marrow: implications for allogeneic hematopoietic stem cell transplantation

**DOI:** 10.1007/s10238-023-01053-w

**Published:** 2023-04-07

**Authors:** Ilaria Laurenzana, Luciana De Luca, Pietro Zoppoli, Giovanni Calice, Alessandro Sgambato, Angelo Michele Carella, Antonella Caivano, Stefania Trino

**Affiliations:** 1 Laboratory of Preclinical and Translational Research, Centro di Riferimento Oncologico della Basilicata (IRCCS CROB), Rionero in Vulture, Italy; 2 Unit of Clinical Pathology, Centro di Riferimento Oncologico della Basilicata (IRCCS CROB), Rionero in Vulture, Italy; 3 Present Address: Department of Molecular Medicine and Health Biotechnology, Università di Napoli Federico II, 80131 Naples, Italy; 4 Scientific Direction, Centro di Riferimento Oncologico della Basilicata (IRCCS CROB), Rionero in Vulture, Italy; 5 Department of Hematology and Stem Cell Transplant Unit, Fondazione IRCCS Casa Sollievo della Sofferenza, San Giovanni Rotondo, Italy; 6 Present Address: Department of Translational Medicine and Surgery, Università Cattolica del Sacro Cuore, Rome, Italy

**Keywords:** Allogeneic hematopoietic stem cell transplantation, Mobilized peripheral blood, Hematopoietic stem/progenitor cells, DNA methylation, Tumor signature, Graft-versus-host disease

## Abstract

Allogeneic hematopoietic stem cell transplantation (AHSCT) is a life-saving treatment for selected hematological malignancies. So far, it remains unclear whether transplanted hematopoietic stem/progenitor cells (HSPCs) undergo epigenetic changes upon engraftment in recipient bone marrow (BM) after AHSCT and whether these changes might be useful in the transplant diagnostics. The purpose of this study was to characterize the whole genome methylation profile of HSPCs following AHSCT. Moreover, the relationship between the observed methylation signature and patient outcome was analyzed. Mobilized peripheral blood (mPB)-HSPCs from seven donors and BM-HSPCs longitudinally collected from transplanted patients with hematological malignancies up to one year from AHSCT (a total of twenty-eight samples) were analyzed using DNA methylation based-arrays. The obtained data showed that DNA methylation of mPB-HSPCs differs between young and adult donors and changes following HSPC engraftment in the BM of recipient patients. Looking at methylation in promoter regions, at 30 days post-AHSCT, BM-HSPCs showed a higher number of differentially methylated genes (DMGs) compared to those of mPB-HSPCs, with a prevalent hyper-methylation. These changes were maintained during all the analyzed time points, and methylation became like the donors after one year from transplant. Functional analysis of these DMGs showed an enrichment in cell adhesion, differentiation and cytokine (interleukin-2, -5 and -7) production and signaling pathways. Of note, DNA methylation analysis allowed to identify a potential “cancer/graft methylation signature” of transplant failure. It was evident in the latest available post-transplant BM-HSPC sample (at 160 days) and surprisingly already in early phase (at 30 days) in patients whose transplant was doomed to fail. Overall, the analysis of HSPC methylation profile could offer useful prognostic information to potentially assess engraftment success and predict graft failure in AHSCT.

**Supplementary Information:**

The online version contains supplementary material available at 10.1007/s10238-023-01053-w.

## Background

Allogeneic hematopoietic stem cell transplantation (AHSCT) is a highly advanced procedure that offers a potential cure for a different number of life-threatening diseases, including hematological malignancies [1]. AHSCT is based on intravenous infusion of hematopoietic stem/progenitor cells (HSPCs) to replace the hematopoietic system after a conditioning treatment. This last one can include myeloablative, non-myeloablative and reduced intensity conditioning and it is essential in eradicating the primary cause of disease, facilitating donor cell engraftment and avoiding transplant rejection via immunosuppression [2]. Allograft stem cell source is mainly represented by bone marrow (BM)- and mobilized peripheral blood (mPB)-derived cells; moreover, an alternative source consists in umbilical cord blood [3, 4]. In general, selection of HSPC origin is closely related to donor availability, patient’s age, disease and other clinical characteristics [5, 6].

However, in the last years, mPB-HSCT significantly increased, accounting for 70% of adult and 30% of children transplants [7], probably due to an easy sourcing of HSPCs, a rapid engraftment of cells and their possible use in non-ablative regimens [4, 8]. Despite the great progress made in recent years including refinement of donor selection, adverse reactions, such as graft-versus-host disease (GvHD), infections and relapse of the underlying disease, still represent a major cause of mortality in transplanted patients [5].

After infusion of donor HSPCs, engraftment is confirmed by gradual rise of both white blood cell and platelet count in PB (generally obtained between 10 and 30 days after transplantation) and by repetitive testing (usually done between 30 and 100 days after transplantation) for donor chimerism in PB/BM [9]. Since the underlying malignant disease is host derived, the decrease of donor chimerism might precede or indicate the imminent relapse, enabling early intervention and presumably better outcome. In this context, a clinical trial is ongoing to demonstrate that analyzing chimerism sooner than 30 days after transplant may help identify problems earlier, get patient treatment sooner and increase the chances of a successful transplant (Clinical Trial NCT03689907) [10]. Recently, several potential biomarkers, ranging from serum proteins and other small molecules to immune cell subsets, have been identified in AHSCT setting [11]. Also, epigenetic changes during transplantation, such as DNA methylation of HSPCs, are being evaluated as potential biomarkers for clinical assessment of AHSCT success.

DNA methylation plays an important role in the dynamic and complex regulation of specific DNA regions, without affecting their sequences [12], making DNA more or less accessible to the transcriptional machinery, and leading gene expression [12]. Epigenetic changes regulate several cellular processes, such as cell development and differentiation. Interestingly, epigenetic signature can be inherited allowing cells to remember their past and alter their behavior [13]. DNA methylation consists in a covalent conversion of cytosine to 5-methylcytosine preferentially found in the context of cytosines adjacent to guanines (CpG) dinucleotides [14, 15]. These last are under-represented in the mammalian genome, accounting for 1% of the whole genome, and are concentrated in large clusters (200 nucleotides or greater) called CpG islands (CGIs), located in the proximity of the transcription start sites (TSSs) of the majority (70%) of human protein-coding genes [12, 16]. It has been observed that impact of DNA methylation on gene expression activation or silencing depends on CpG genomic location. In particular, while CGI promoter methylation is associated with stable gene silencing, the regulatory role of DNA methylation outside CGIs in ‘shores’ and ‘shelves’ and throughout gene bodies is less extensively studied [12, 17].

It remains unclear whether epigenetic changes of donor HSPCs are recapitulated upon engraftment into recipient BM. In a previous work, we showed that donor BM-HSPCs after engraftment in BM of patients with hematological malignancies displayed significant changes in the global methylation profile with prevailing hyper-methylation, suggesting an initial genome silencing which decreased within 60 days up to one year. Moreover, our preliminary data also suggested that the methylation profile could be used as predictor of relapse [18].

Here, we analyzed the methylation profile of mPB-HSPCs from donors and of BM-HSPCs post-AHSCT to provide an insight of its dynamic/evolution and to evaluate whether analysis of HSPC methylation profile could help in monitoring the transplant success.

## Methods

### Study design and donor and patient samples

A total of 7 donors and their respective 7 recipients who received mPB-AHSCT between 2013 and 2015, followed-up until August 2022, were included in our study. Granulocyte colony-stimulating factor (G-CSF) mobilized-donor PB samples (indicated as T0) and BM aspirates of recipients were provided by the Department of Hematology and Stem Cell Transplantation Unit, IRCCS “Casa Sollievo della Sofferenza” Hospital, San Giovanni Rotondo, Italy. Transplanted patients were affected by acute myeloid leukemia (AML; n = 3), acute lymphoblastic leukemia (ALL; n = 1), chronic lymphocytic leukemia (CLL; n = 1), multiple myeloma (MM; n = 1) and Hodgkin lymphoma (HL; n = 1). Collection of sequential recipient specimens at different time points after transplant, day + 30 (T1), + 60 (T2), + 120 (T3), + 180 (T4) and + 365 (T5), was performed. For each time point, the following number of samples was available: n = 6 for T1, n = 2 for T2, n = 2 for T3, n = 7 for T4 and n = 4 for T5. Both patient and donor characteristics are reported in Table 1. Engraftment (days) is defined as the first of three consecutive days with more than 0.5 × 10^9^/L neutrophils and untransfused platelet count greater than 20 × 10^9^/L in PB. The presence of chimerism was evaluated after transplantation in PB by genomic polymorphism analysis.

**Table 1:** Characteristics of donors and patients subjected to allogeneic hematopoietic stem cell transplantation

Patient n.	Patient Age/Sex	Diagnosis	Conditioning regimen	Donor type	Donor Age/Sex	Time points post-AHSCT (days)	Disease state post-AHSCT	Engraftment (days)	Chimerism	Immuno suppressive agents, duration (months)	Follow-up^a^
P3	30/2	AML	Myeloablative	MUD	23/2	T1 = 34	CR	N = + 14 P = + 12	100%	CSA, MTX, ATG (6)	**DFS:** + 7 months post AHSCT; **OS**: + 8 months post AHSCT; **R**: no; **NRM:** no; **aGvHD:** yes, oral cavity, + 3 months post AHSCT; **cGvHD**: no; **GvHDf RfS**: 0
						T4 = 162	CR		100%		
P5	35/2	HL	RIC	MUD	40/2	T1 = 36	CR	N = + 17 P = + 15	100%	CSA, MTX, ATG (6)	**DFS/OS:** 111 months post AHSCT; **R**: no; **NRM:** no; **aGvHD:** no; **cGvHD:** no; **GvHDf RfS:** no
						T3 = 122	CR		100%		
						T4 = 180	CR		100%		
						T5 = 360	CR		100%		
P7	61/2	AML	Myeloablative	MUD	19/2	T2 = 47	CR	N = + 17 P = + 14	100%	CSA, MTX, ATG (6)	**DFS/OS:** 107 months post AHSCT; **R**: no; **NRM**: no; **aGvHD**: no; **cGvHD**: no; **GvHDf RfS:** no
						T4 = 180	CR		100%		
P8	62/2	CLL	RIC	MUD	40/2	T1 = 26	CR	N = + 24 P = + 30	100%	/	**DFS/OS:** 110 months post AHSCT; **R:** no; **NRM:** no; **aGvHD:** no; **cGvHD:** no; **GvHDf RfS:** no
						T4 = 180	CR		100%		
						T5 = 351	CR		100%		
P10	53/2	ALL	Myeloablative	MUD	31/2	T1 = 30	CR	N = + 14 P = + 13	100%	CSA, MTX, ATG (6)	**DFS/OS:** 106 months post AHSCT; **R:** no; **NRM:** no; **aGvHD:** no; **cGvHD**: no; **GvHDf RfS**: no
						T2 = 58	CR		100%		
						T4 = 193	CR		100%		
						T5 = 362	CR		100%		
P14	54/1	MM	RIC	MSD	49/1	T1 = 30	PR	N = + 13 P = + 12	96%	CSA (5), MTX (1)	**DFS:** 0 months; OS**:** 10 months post AHSCT;** R**: no; **NRM:** yes (CMV reactivation) **aGvHD**: no; **cGvHD:** no; **GvHDf RfS**: no
						T4 = 163	PR		93%		
P15	44/1	AML	Myeloablative	MUD	32/2	T1 = 27	CR	N = + 11 P = + 13	100%	CSA, MTX, ATG (6)	**DFS/OS:** 103 months post AHSCT; **NMR:** no; **aGvHD**: no; **cGvHD:** no; **GvHDf RfS**: no
						T3 = 115	CR		100%		
						T4 = 178	CR		100%		
						T5 = 375	CR		100%		

^a^Follow-up to August 2022

*AHSCT* allogeneic hematopoietic stem cell transplantation; sex = 1 or 2, *AML* acute myeloid leukemia, *HL* Hodgkin lymphoma, *CLL* chronic lymphocytic leukemia, *ALL* acute lymphoblastic leukemia, *MM* multiple myeloma, *RIC* reduced intensity conditioning, *MUD* matched unrelated donor, *MSD* Matched sibling donor, *CR* complete remission, *PR* partial remission, *CSA* cyclosporine, *MTX* methotrexate, *ATG* anti-thymocyte globulin, *DFS* disease-free survival, *OS* overall survival, *R* relapse, *NRM* non-relapse mortality, *aGvHD* acute graft versus host disease, *cGvHD* chronic GvHD, *GvHDf RfS* GvHD-free relapse-free survival, *CMV* cytomegalovirus. Engraftment days = days in which patients achieved an absolute neutrophil (N) and platelet (P) count of ≥ 500/μl and > 20,000/μl, respectively

All patients gave informed consent in accordance with the Declaration of Helsinki and the study was approved by IRCCS CROB.

### Human CD34^+^ HSPC isolation

Both donor mPB and patient BM mononuclear cells were obtained by Ficoll-Paque gradient centrifugation. CD34^+^ cells were isolated from mononuclear cells by CD34 Microbead Kit (Miltenyi Biotec, Auburn, CA), as previously reported [18]. Their purity, evaluated by flow cytometry, ranged between 90 and 95%.

### Genomic DNA isolation

DNA was extracted from CD34^+^ cells by AllPrep DNA/RNA Micro Kit (Qiagen GmbH, Hilden, Germany) following manufacturer's instructions. DNA quality was controlled by agarose gel electrophoresis and quantified by a NanoDrop ND-1000 Spectrometer (Thermo Scientific, Wilmington, DE, USA).

### Bisulfite conversion and array-based DNA methylation

Genomic DNA (250 ng) was treated with sodium bisulfite using the Zymo EZ DNA Methylation Kit (Zymo Research, Orange, CA, USA) according to the manufacturer’s instructions, with the alternative incubation conditions recommended for the Illumina Infinium Methylation Assay (Illumina, CA, USA). Array-based DNA methylation was performed according to the Infinium HD Methylation Assay protocol and Infinium Human Methylation EPIC (850 k) BeadChip (Illumina), as previously described [18]. BeadChips were scanned using the IlluminaHiScanSQ system (Illumina).

### Microarray data analysis

Illumina Infinium Human Methylation EPIC idat files were imported into R/Bioconductor according to Todoerti et al. [19]. Methylation level for each cytosine was expressed as β-value (ratio of fluorescence intensity of methylated probes vs all probes) as well as M-value (log2 ratio of the intensities of methylated probe versus unmethylated probe). Although β-value has a more intuitive biological interpretation, M-value is more statistically valid [20] so, β-value was used for description and plotting and M-value for statistical analysis. A β-value of 0 represents an unmethylated CpG site and a β-value approaching 1 represents a fully methylated CpG site. As thresholds to define hyper- and hypo-methylation, it was proposed 0.2 and 0.8, respectively, with the intervening values indicative of intermediate levels of methylation [20].

Overlap analysis of methylation EPIC probes with genomic features (such as TSS1500, TSS200, 1st Exon, 5’UTR, Gene body, 3’UTR and IGR) and with CpG localization (Islands, Shores, Shelves or Open Sea) was determined exploiting the annotation stored in the Illumina’s EPIC methylation arrays Bioconductor package [21]. Promoter included TSS200, TSS1500, 5’UTR, and body regions included 1st exon, gene body, 3’UTR, exon boundaries. According to the distance from CpG islands, CpG shores were localized up to 2 kb away from CGIs, CpG shelves from 2 to 4 kb from CGIs and open sea at > 4 kb away from CGIs.

Principal Component Analysis (PCA) was performed based on principal components 1 (on x-axis) and 2 (on y-axis) that were plotted to visualize pattern of relationship among samples. Clustering analysis was performed on the methylation level (M-value) of the most variable probes.

For each time point, we also evaluated methylation median level of promoter and gene body regions. Differential methylation analysis was performed using limma, minfi and DMRcate packages [22–24]. Differentially methylated probes (DMPs) and differential methylated regions (DMRs) specifically annotated for gene region and CGI position and their relative distribution (as probes, genes and regions) were analyzed. Probes with absolute FC (M-value) greater than 1.5 (log2(M-value) > 0.58) and with *p*-value < 0.05 were considered significant.

On the bases of DMPs referred to promoter region, we obtained the lists of differentially methylated genes (DMGs) (*p*-value < 0.05). Subsequently, we used these lists for enrichment analysis and significant results (adjusted *p*-value < 0.05) among the mSigDB genes sets were obtained exploiting clusterProfiler package [25, 26].

To better understand stable methylation modifications in the promoter area, we defined genes significantly and differentially methylated in T1 versus T0 and T4 versus T0 as “stable” while the discordant genes were labeled as “revert”. We also highlighted the genes “stable” at T2 vs T0.

All analyses were performed using R software [27] and CRAN/Bioconductor packages (https://www.r-project.org/; https://www.bioconductor.org/).

## Results

### Genome-wide methylation pattern in donor and recipient HSPCs

**Fig. 1:**
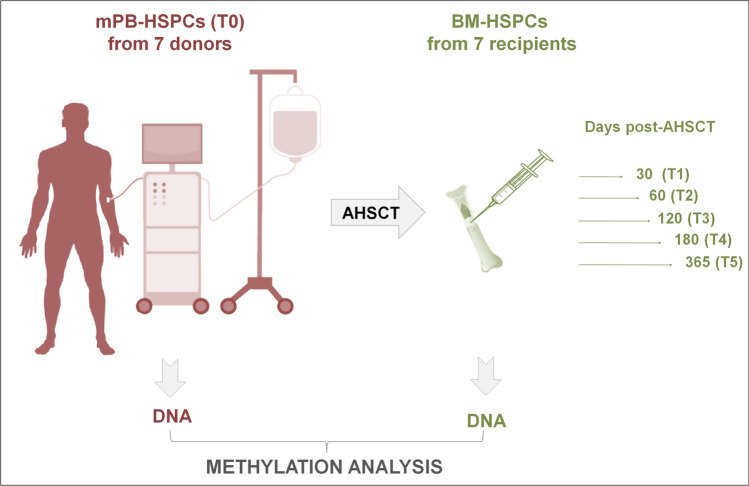
Experimental design. DNA methylation analyzed in mobilized PB (mPB)-HSPCs of 7 donors (T0) and in BM-HSPCs of 7 recipients at the following time points after transplant: + 30 (T1), + 60 (T2), + 120 (T3), + 180 (T4) and + 365 (T5) days

In order to explore DNA methylation pattern in allogeneic transplantation, we analyzed genome wide methylation profile of G-CSF mPB-HSPCs from seven donors and HSPCs from BM of respective recipients, longitudinally followed, after transplant up to one year (now referred as T1: + 30, T2: + 60, T3: + 120, T4: + 180, T5: + 365 days). Table 1 summarizes the information of donors and patients. In Fig. 1 we reported a graphical representation of this study. Specifically, we interrogated over 850,000 methylation sites distributed across the genome of 28 HSPC samples. Principal component analysis (PCA) performed on donor and patient HSPCs was constructed based on probes with largest standard deviation among samples to identify possible confounding factors (Supplementary Figure S1a–d). PCA plots reporting donor samples (T0) and recipients in all time points (T1–T5) (Supplementary Figure S1a), or disease of patients (Supplementary Figure S1b) showed that DNA methylation was uniformly distributed among all samples and no confounding factors were found.

**Fig. 2:**
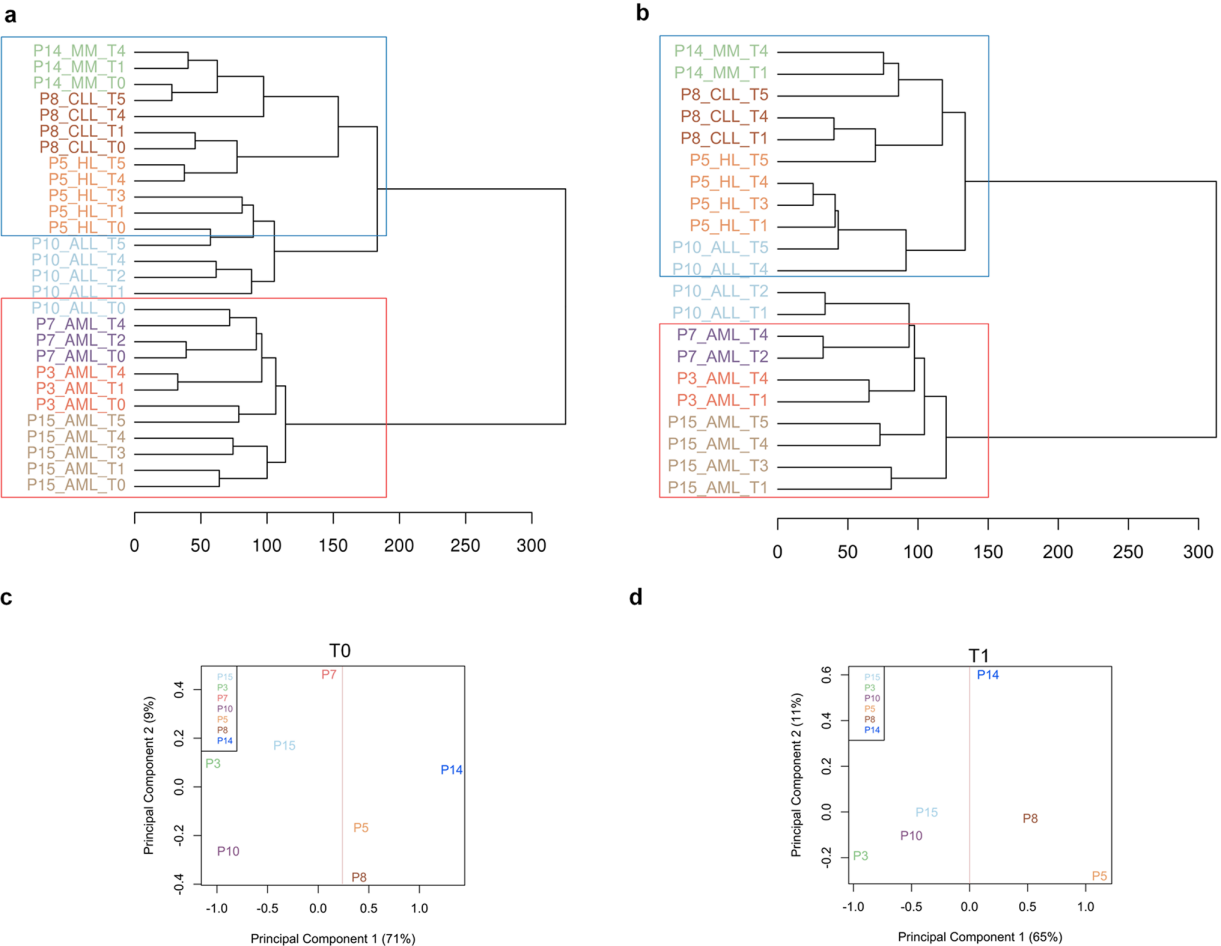
Genome-wide methylation pattern in donor and recipient HSPCs. **a, b** Unsupervised hierarchical clustering of global HSPC methylation profile on the most variable probes for all samples with (**a**) and without donors (**b**). Each sample is indicated as patient (P) number, disease (MM, CLL, HL, ALL and AML) and time points (T). Donor HSPCs are indicated as P14_MM_T0, P8_CLL_T0, P5_HL_T0, P10_ALL_T0, P7_AML_T0, P3_AML_T0, and P15_AML_T0. Recipient HSPCs at different time points (T1–T5) are indicated as P14_MM_T1, P8_CLL_T1, P5_HL_T1, P10_ALL_T1, etc. Each patient with its donor is annotated with a specific color. The blue and red rectangles indicate two different clusters. **c, d** Multidimensional scaling plot of **c** donors (T0) and **d** T1 patient samples. Samples are annotated by patient (P) number and are divided into two clusters by red line (Color figure online)

Unsupervised hierarchical clustering of global methylation profile of 28 samples performed on the most variable probes showed two main clusters (Fig. 2a). Specifically, one cluster contained HSPCs from donors of P14 (P14_MM_T0), P8 (P8_CLL_T0) and P5 (P5_HL_T0; now referred as adults: age ≥ 40 years), while the second one included HSPCs from donors of P15 (P15_AML_T0), P3 (P3_AML_T0), P7 (P7_AML_T0) and P10 (P10_ALL_T0; now referred as young: age ≤ 33 years). In addition, in each cluster, HSPCs of each donor (T0) and those of their respective time points (T1-T5) after transplant grouped close together, while HSPCs in T1-T5 of P10 did not cluster with their donor HSPCs (Fig. 2a).

Furthermore, looking only at HSPCs from recipient patients in the different time points (n = 21 samples) without donor samples, a distribution similar to that obtained in the first unsupervised hierarchical clustering was observed (Fig. 2b). In particular, BM-HSPCs from P14, P8, P5 and P10 patients (MM, CLL, HL and ALL, respectively) clustered together, while those from P15, P3 and P7 patients (AML) grouped separately in a second cluster and included T1 and T2 of P10.

PCA of only donor HSPCs showed their separation into two groups on principal component 1 (x-axis); specifically, one cluster with P3, P7, P10 and P15 (young donors) and another with P5, P8 and P14 (adult donors) were observed. P7 donor was distant on principal component 2 (y-axis) respect to other samples (Fig. 2c). In PCA, HSPCs from recipient patients at T1 were distributed into two groups similarly to those observed for donor subjects. P14 T1 segregated separately from other samples on principal component 2 (Fig. 2d).

To verify the potential similarities or differences of methylation profile among donors (T0) and patient sequential time points (T1-T5), we evaluated the global methylation level, expressed by β-value, in each group (Supplementary Figure S1c-d). This analysis revealed a similar hypo-methylation median level in all samples (T0-T5) (Supplementary Figure S1c), and a prevalent hypo-methylation in promoter and hyper-methylation in gene body regions in each time point group (Supplementary Figure S1d).

### Changes in DNA methylation after transplant

**Fig. 3:**
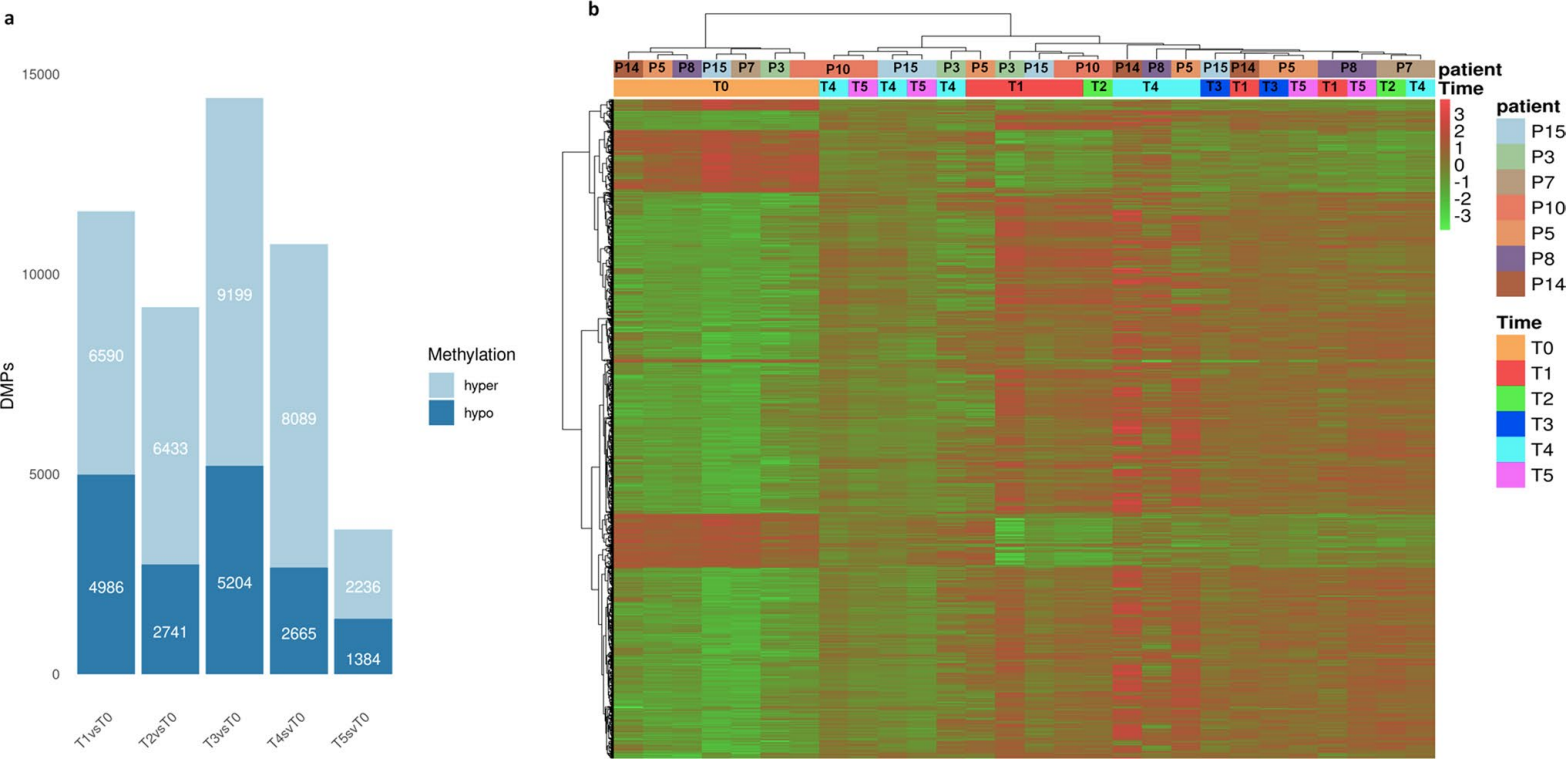
Methylation profile of donor and recipient HSPCs. **a** Number of DMPs at each recipient time point vs donors (T1vsT0, T2vsT0, T3vsT0, T4vsT0 and T5vsT0) distinct in hypo- and hyper-methylated probes (dark and light blue, respectively). **b** Heatmap of significant common donor (T0) and recipient (T1–T5) DMPs by hierarchical clustering analysis. DNA methylation levels are displayed as Z-score by color-coded and represented in a color scale from green (hypo-methylation) to red (hyper-methylation), as indicated at the right. Each column represents a donor or a patient at a specific time point. Columns are ordered according to performed clustering (Color figure online)

In order to verify methylation changes of HSPCs after transplant, firstly we performed a differential analysis between all grouped T0 vs all grouped post-AHSCT time points obtaining differentially methylated probes (DMPs) (Fig. 3a, Supplementary Table S1). We observed an elevated number of DMPs in T1 compared to T0 (T1vsT0); specifically, 11576 DMPs (6590 hyper- and 4986 hypo-methylated) were found. DMP differences were observed in the other comparisons too, with 9174 DMPs (6433 hyper- and 2741 hypo-methylated) in T2, 14403 DMPs (9199 hyper- and 5204 hypo-methylated) in T3 and 10754 DMPs (8089 hyper- and 2665 hypo-methylated) in T4vsT0. Conversely, it was identified a marked reduction of DMPs in T5 (T5vsT0) respect to T1vsT0 reaching a number of 3620 (2236 hyper- and 1384 hypo-methylated). In addition, we observed a greater hyper-methylation of DMPs in all time points vs T0, more evident in T2vsT0 and T4vsT0 (Fig. 3a). In addition, DMPs from T1 and T4 vs T0 were hyper-methylated in a range of 57–75% and hypo-methylated in a range of 25–43% (Fig. 3a).

We also analyzed differentially methylated regions (DMRs) containing single or clusters of DMPs (Supplementary Figure S2a, Supplementary Table S2). Similarly to DMPs, an elevated number of DMRs in T1 versus T0 (T1vsT0; n = 605, of which 474 hyper- and 131 hypo-methylated) was detected and this difference was maintained in T4 (T4vsT0; n = 644, of which 523 hyper- and 121 hypo-methylated DMRs), while DMRs were not observable in T2 and T3, probably due to a lower number of samples in these time points (n = 2 samples for T2 and T3).

Secondly, we compared methylation level of all common significant DMPs between all samples at T1, T2, T3, T4 and T5 vs those at T0.

As shown in Fig. 3b, hierarchical clustering showed different methylation profiles between donors (on the left of the heat map) and recipients (on the right of the heat map) with a prevalent hypo-methylation in donor cells and a hyper-methylation in HSPCs after transplant. Looking at donors, two clusters could be distinguished: one including P14, P5 and P8 donors and another with P15, P7, P3 and P10 donors. Observing HSPCs longitudinal time points (to the right of the heat map), it was visible a separation into two groups: one, closer to donors, including T4 and T5 of P10 and P15, and T4 of P3, and a second one with all other samples. In this last cluster, hyper-methylation was prevalent, while in the first one (closer to donors) a reduced hyper-methylation was observed in favor of hypo-methylation (Fig. 3b). Of note, in the second cluster, we noted one group with T1 of P3, P15 and P10 together with its T2, another with T4 of P14, P8 and P5, while T1 of P14 and P8 were separated from others T1. In addition, the median β-value was reported in a Supplementary Table S3.

All these data indicated an “global upheaval” of donor HSPC methylation at 30 days (T1) that lasts up to 180 (T4) days after transplantation and reverses after one year (T5).

To better describe DMPs resulting from comparison of recipients at each time point vs donors, we investigated their localization in promoter or in body regions and in CpG sites (Island, OpenSea, Shelf and Shore) (Supplementary Figure S2b, Supplementary Table S1). In particular, we found that all regions were more hyper- than hypo-methylated in both promoter and gene body regions, except for Shore localized in body that resulted more hypo-methylated in T4 vs T0. Moreover, in T5 vs T0 comparison, hypo- and hyper-methylated probes showed a similar level in both shelf and shore of promoter, as well as in shore of body region. Of note, open sea in both promoter and body regions resulted more differentially methylated respect to the other regions (Supplementary Figure S2b).

### Gene signature after transplant

**Fig. 4:**
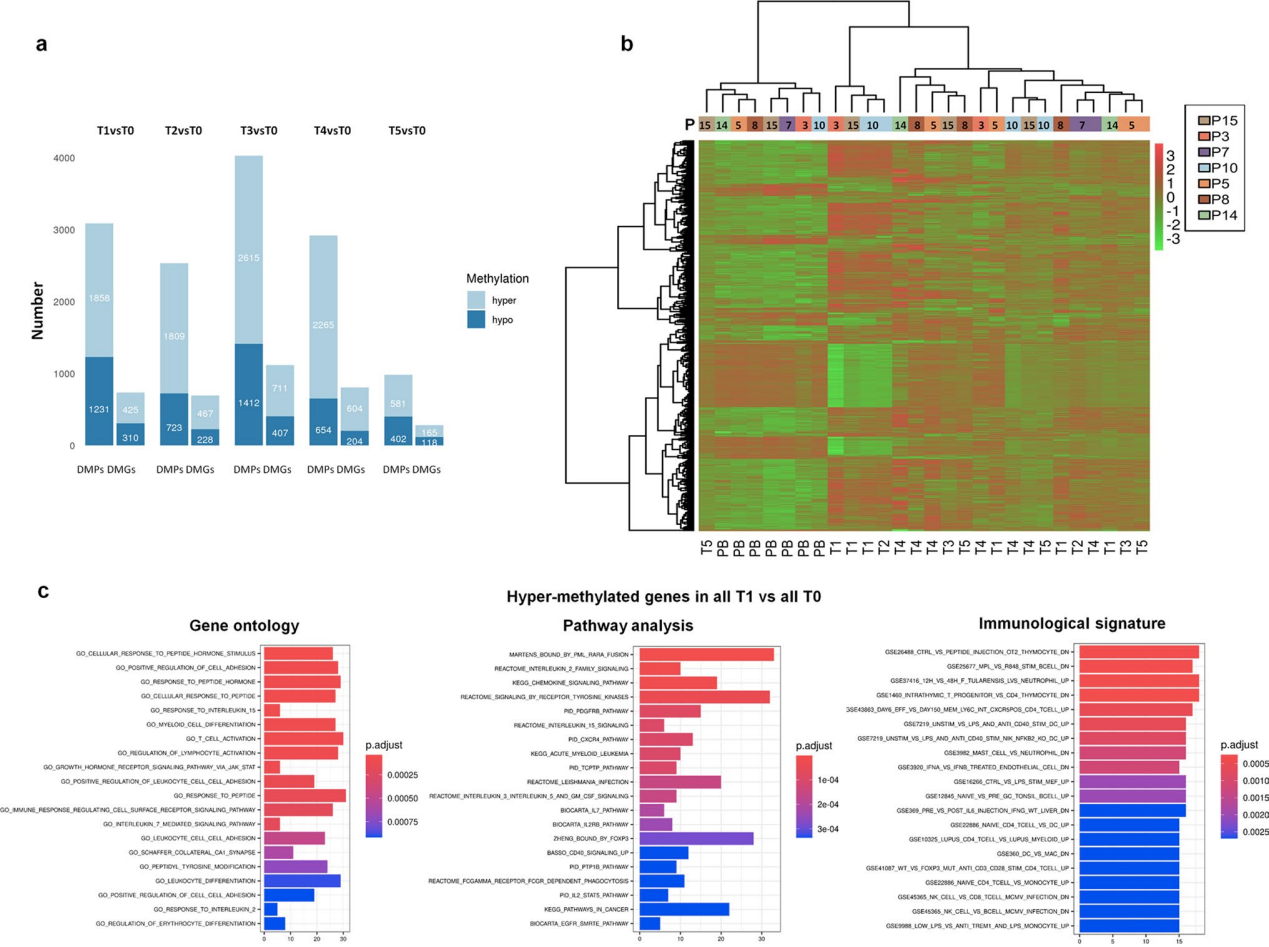
Differential methylated gene profile during transplant. **a** Number of DMPs located in promoter and respective DMGs at each recipient time point vs donors (T1vsT0, T2vsT0, T3vsT0, T4vsT0 and T5vsT0). **b** Heatmap of all significant common DMGs between donors and recipients by unsupervised analysis. DNA methylation levels are displayed as Z-score by color-coded and represented in a color scale from green (hypo-methylation) to red (hyper-methylation), as indicated at the right. Each column represents a donor (PB) or a patient at a specific time point (T1, T2, T3, T4 and T5) as reported on the bottom of heatmap. Columns are ordered according to performed clustering. **c** Gene ontology (GO), pathway analysis and immunological signature of hyper-methylated genes obtained by comparison of all T1 with all T0. The *y*-axis shows GO, pathways or immunological signature and the *x*-axis represents the number of enriched genes. The color scale shows the significance of each GO, pathway analysis or immunological signature showing the − log_10_ (adjusted *p*-value), with smaller *p*-value (red) representing more significant enrichment (Color figure online)

To obtain a gene signature of HSPCs after transplant, we analyzed DMPs in the promoter region of all patient time points (T1-T5) compared to donors (T0) and then we converted them in their respective DMGs (Fig. 4a, Supplementary Table S1 and S4).

Similarly to global DMPs, firstly, we found a higher number of both promoter DMPs and DMGs in T1 vs T0 comparison (T1vsT0). This difference was maintained in the other comparisons (T1–T4 vs T0), although with a slight perturbation in T2 and T3 time points only for DMPs. Specifically, we obtained in T1vsT0 a list of 3089 DMPs corresponding to 735 genes, 2532 DMPs/695 genes in T2, 4027 DMPs/1118 genes in T3 and 2919 DMPs/808 genes in T4vsT0. Instead, a marked reduction of DMPs/DMGs was observed at the last patient time point (T5vsT0) respect to T1vsT0, reaching 983 DMPs/283 genes (Fig. 4a). Moreover, a prevalent hyper-methylation (about 60%) compared to hypo-methylation was found in all comparisons for both promoter DMPs and DMGs (Fig. 4a). Hyper-methylation increased passing from T1vsT0 (57%) to T2–T4vsT0 (65–74%) and reduced in T5vsT0 (58%) (Fig. 4a). The unsupervised analysis of all samples looking at union of DMGs of donors vs different time samples of the recipients showed two main clusters: one with donor HSPCs (on the left of the heat map and indicated as PB) in which the majority of genes were hypo-methylated and the second one with all the post-AHSCT time points (Fig. 4b). Looking at donor cluster, it was possible to separate P3/P7/P10/P15 (young) from P5/P8/P14 (adult) HSPC donors. Observing the other time points, two main clusters were evident: one including T1 of P3, P15 and P10, with a prevalence of hyper-methylated genes and another one with the remaining time points with a lower level of hyper-methylation respect to that observed in T1 cluster. In particular, in this last one, we observed that T1 of P5, P8 and P14 were separated from each other; T4 of P14/P8/P5 and that of P3/P10/P15 formed two neighboring clusters (Fig. 4b). In addition, the median β-value was reported in a Supplementary Table S5.

To understand which functions were associated with methylation changes already observed at one month from transplant (T1), we performed a functional analysis of the hyper- and hypo-methylated genes obtained in the comparison of all T1 versus all T0 (Fig. 4c and Supplementary Figure S3, Supplementary Table S6). In particular, gene ontology (GO) analysis of hyper-methylated genes identified (*i*) generic pathways involved in regulation of adhesion (GO_positive_regulation_of_cell_adhesion), response to interleukins (IL-) such as IL-15, IL-7 and IL-2 (GO_response_to_interleukin_15, GO_response_to_interleukin_2, Go_response_to_interleukin_7), regulation of hematopoiesis, activation (GO_positive_regulation_of_cell_activation), and in change of state or activity of cell, such as movement, cytokine secretion and (*ii*) pathways of regulation/activation of specific cell populations such as myeloid cells (GO_myeloid_cell_differentiation), lymphocyte and erythrocytes (GO_regulation_of_lymphocyte_activation, GO_positive_regulation_of_erythrocyte_differentiation) (Fig. 4c). In addition, hyper-methylated genes, evaluated for pathway analysis, were enriched for several categories, such as IL-2, IL-3, IL-5, IL-15 (e.g. Reactome_interleukin_2_family_signaling), granulocyte–macrophage colony-stimulating factor (GM-CSF) and chemokine signaling, C-X-C motif chemokine receptor 4 (CXCR4; Pid_CXCR4_pathway) and IL-2/Signal transducer and activator of transcription 5 (STAT5) pathways (KEGG_JAK_STAT_signaling_pathway), involved in hematopoietic homeostasis, growth, trafficking and immune function (Fig. 4c). We also found an enrichment for tumor pathways such as acute and chronic myeloid leukemia (KEGG_acute_myeloid_leukemia, KEGG_chronic_myeloid_leukemia), lymphoma (Pasqualucci_lymphoma_by_gc_stage_dn) and multiple myeloma (Zhan_multiple_myeloma_lb_dn). Moreover, immunological signature of hyper-methylated genes showed a myeloid (e.g., monocytes and mast cells) and lymphoid (e.g., CD4 T cells and B cells) modulation of various immune-cell functions (e.g., GSE22886_naive_CD4_Tcell_vs_monocyte_up) (Fig. 4c). Functional analysis of hypo-methylated genes enriched in pathways and immunological signature involved in lymphocyte development and activation (e.g., KEGG_B_cell_receptor_signaling_pathway) (Supplementary Figure S3a).

Subsequently, we verified which methylation changes occurred at 30 days (T1), in terms of DMGs, remained modified at 180 days (T4). Therefore, DMGs of T1 vs T0 were crossed with DMGs of T4 vs T0 and the common DMGs were identified. These last DMGs were indicated as “stable genes”, while the other ones were defined “reverted genes” (Supplementary Table S7). We annotated 178 DMGs (n = 158 hyper- and n = 20 hypo-methylated) that remained modified up to 180 days (T4), showing the same methylation status from 30 days (T1) up to 180 days (T4) after transplant, while 391 DMGs (n = 306 hyper- and n = 85 hypo-methylated) resulted as “reverted genes”. GO and pathways analysis of hyper-methylated “stable genes” showed their involvement in modulation of different physiological cell functions, such as response of receptor tyrosine kinase, PDGFR pathways, inflammation control, homeostatic regulation, tissue development, trafficking and in the change of state or activity of cells. Moreover, immunological signature revealed that hyper-methylated “stable genes” were significantly enriched in leukocyte immune modulation (Supplementary Table S8, Supplementary Figure S3b). The hypo-methylated “stable genes” were too few for any type of enrichment. At T5, instead, we observed a remarkable reduction of “stable genes”. In fact, only 9 genes remained modified until T5 and, specifically, 3 hypo- (*SLC2A9, TRIM15* and *SIPA1L1*) and 6 hyper-methylated (*PRKAR1B, TEK, MRVI1, KCTD11, SGK494* and *KCNJ16*) genes (Supplementary Table S9).

Collectively, we observed that all donor HSPCs showed a similar gene methylation that changed when they engrafted in recipient BM and then returned as at the starting point. Changing genes were involved in hematopoietic homeostasis.

### Association of methylation data with clinical outcome of transplanted patients

In order to explore a possible “prediction power” of HSPC methylome profile in failure AHSCT, we analyzed data from two patients who died after transplant. The first patient, indicated as P14, which was affected by MM, received mPB-HSPCs post RIC in partial remission, and died 290 days after AHSCT for reactivation of human cytomegalovirus (HCMV) infection. The last available time point of BM-HSPCs (T4 = 163 days) was collected 127 days before patient death.

**Fig. 5:**
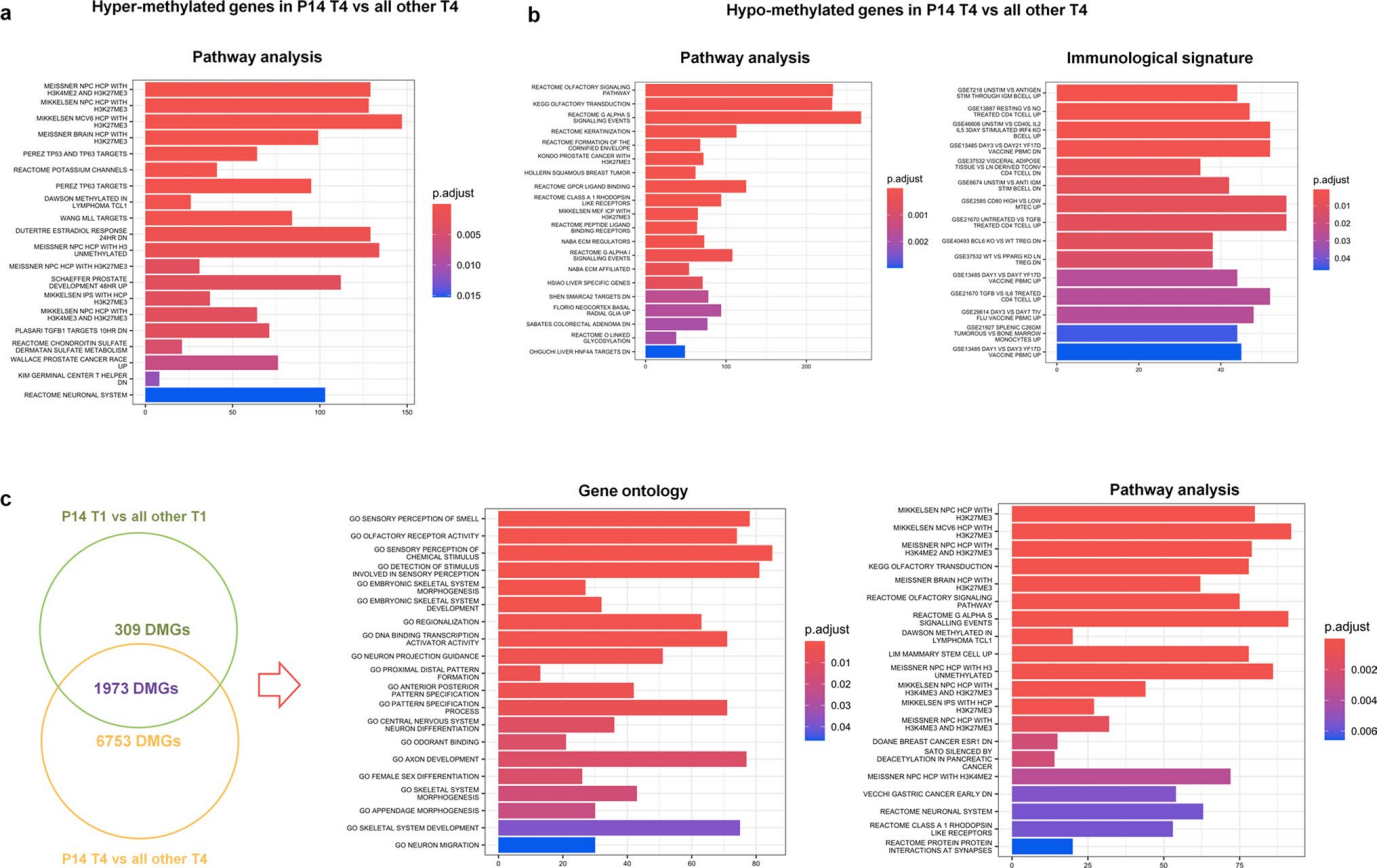
Functional analysis of DMGs obtained from the comparison of P14 T4 with all other patient T4. **a** Pathway analysis of hyper-methylated genes and **b** pathway analysis and immunological signature of hypo-methylated genes obtained by comparison of P14 T4 vs all other T4. **c** Gene signature of DMGs obtained crossing P14 T4-vs-all T4 comparison with P14 T1 vs all T1 comparison. Venn diagram shows the number of common DMGs (n = 1973) between the two comparisons and the non-overlapping numbers (n = 6753 and n = 309) specifying the unique genes to each condition: P14 T4-vs-all T4 and P14 T1 vs all T1. GO and pathway analysis of hyper- and hypo-methylated common DMGs (n = 1973) between the two comparisons, P14 T4 vs all T4 and P14 T1 vs all T1 are represented. The *y*-axis shows pathway analysis, immunological signature and GO, and the *x*-axis represents the number of enriched genes. The color scale shows the significance of each GO or pathway showing the − log_10_ (adjusted *p*-value) with smaller *p*-value (red) representing more significant enrichment (Color figure online)

We evaluated the last available time point T4 (+ 163 days) of P14 observing a more remarkable different methylation pattern of HSPCs respect to those of the same time point of all live patients (P5, P7, P8, P10, P15), corresponding to 8726 DMGs (4720 hyper- and 4006 hypo-methylated) (Supplementary Table S10A). GO functional analysis of these DMGs showed, for hyper-methylated genes, an enrichment of processes related to cell specification and commitment (e.g., GO_Pattern_Specification_Process, GO_Cell_Fate_Commitment), and correlated with transcriptional activity (e.g., GO_DNA_Binding_Transcription_Activator_Activity, GO_DNA_Binding_Transcription_Repressor_Activity) (Supplementary Figure S4a, Supplementary Table S11). Pathway analysis revealed modulation of DNA methylation and histone modifications; in addition, pathways involving transcription factors that regulate key cellular processes were also found (e.g., Perez_TP53_and_TP63_targets) and a significant enrichment directly connected with tumors such as lymphoma (Dawson_Methylated_in_Lymphoma_TCL1), prostate and bladder cancer (e.g., Wallace_Prostate_Cancer_Race_UP, Lindgren_Bladder_Cancer_Cluster_2B) was also observed (Fig. 5a).

GO of hypo-methylated genes showed generic cell functions including intermediate filament of cytoskeleton, extracellular matrix and G protein signaling pathways (Supplementary Figure S4b; Supplementary Table S11). In pathway analysis, different tumor pathways such as breast, prostate and gastric cancers were highlighted (Fig. 5b). Finally, for immunological signature, we identified genes involved in CD4 T lymphocytes and TGF-β modulation (e.g., GSE13887_resting_vs_no_treated_CD4_Tcell_up, GSE21670_TGFβ_vs IL6_treated_CD4_Tcell_up) (Fig. 5b).

In order to understand whether DMGs of P14, found at 163 days, were already present at 30 days post transplantation (T1), we firstly verified the methylation profile of P14 in the first available time point after transplantation (T1) compared to the T1 of all the other live patients by finding 2282 DMGs (1110 hyper- and 1172 hypo-methylated) (Supplementary Table S10b). Their enrichment analysis in GO/pathway/immune signature identified (*i*) generic pathways involved in transcriptional activation and cell fate specification, and (*ii*) tumor pathways such as lymphoma, lung, breast and prostate cancers (Supplementary Table S12, Supplementary Figure S4c–d).

As second step, we intersected DMGs of P14 T4 vs all T4 with those of P14 T1 vs all T1 and we found 1973 common DMGs (Fig. 5c, Supplementary Table S10). Interestingly, these DMGs showed the same methylation pattern (hyper- or hypo-methylation) with an increased methylation level passing from P14 T1 vs all T1 to P14 T4 vs all T4. Functional analysis of those DMGs revealed modulation of generic functions for GO, including transcription activity; notably, pathway analysis showed their participation in histone modifications and tumor pathways (Fig. 5c, Supplementary Table S13).

Subsequently, we analyzed HSPC methylation of another patient, P3, affected by AML who died 243 days after transplant. The last available time point of BM-HSPCs (T4 = + 162 days) was collected 81 days before patient death occurred for disease progression.

**Fig. 6:**
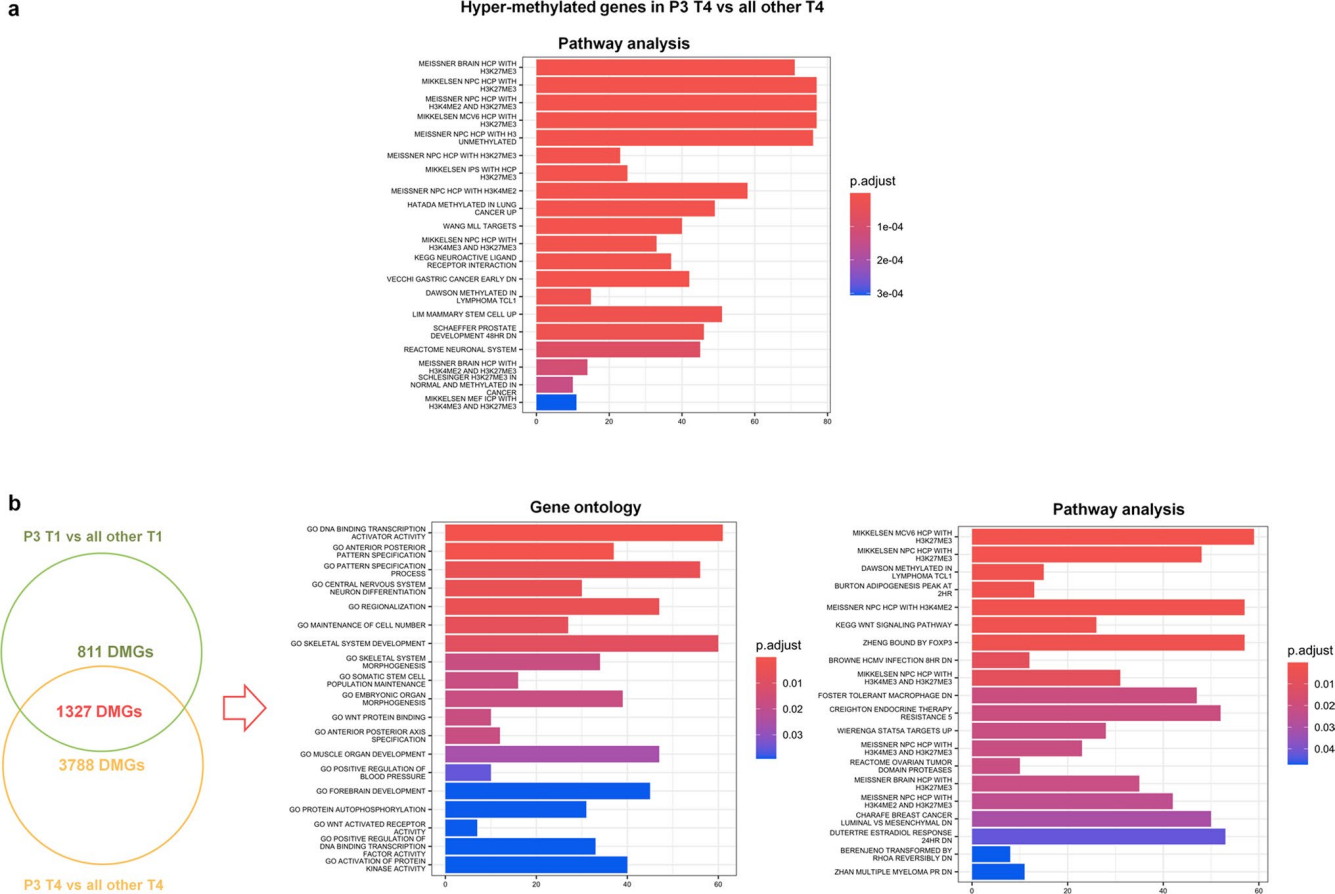
Functional analysis of DMGs obtained from the comparison of P3 T4 vs all other patient T4. **a** Pathway analysis of hyper-methylated genes obtained by comparison of P3 T4 vs all other T4. **b** Gene signature of DMGs obtained crossing P3 T4 vs all T4 comparison with P3 T1 vs all T1 comparison. Venn diagram shows the number of common DMGs (n = 1327) between the two comparisons and the non-overlapping numbers (n = 3788 and n = 811) specifying the unique genes to each condition: P3 T4 vs all T4 and P3 T1 vs all T1. GO and pathway analysis of hyper- and hypo-methylated common DMGs (n = 1327) between the two comparisons, P3 T4 vs all T4 and P3 T1 vs all T1 are represented. The *y*-axis shows pathway and GO analysis, and the *x*-axis represents the number of enriched genes. The color scale shows the significance of each GO or pathways showing the − log_10_ (adjusted *p*-value) with smaller *p*-value (red) representing more significant enrichment (Color figure online)

Similarly, to P14, we compared methylation of this last time point, T4 (+ 162 days), with that of T4 from live patients, finding 5115 DMGs (2014 hyper- and 3101 hypo-methylated) (Fig. 6, Supplementary Table S14a).

Enrichment analysis of hyper-methylated genes showed the GO with transcriptional activity, cell fate commitment, cell signaling, stem cell differentiation and proliferation (Supplementary Figure S5a, Supplementary Table S15). Interestingly, pathway analysis identified many tumor pathways (e.g., Hatada_Methylated_in_lung-cancer, Vecchi_Gastric_Cancer_early_DN, Dawson_Methylated_in_Lymphoma_TCL1, Rickman_Head_and_Neck_Cancer_A) including mixed lineage leukemia (MLL) pathway (Wang_MLL_Targets) and GvHD (KEGG_Graft_versus_Host_Disease; Fig. 6a). In addition, we found processes related to cell specification/commitment, modulation of DNA methylation and histone modifications in cancer. Immunological profile mostly identified genes related to plasma cells and B cells (Supplementary Figure S5a).

In order to understand if DMGs of P3 at 162 days (T4) were already present at 32 days post transplantation (T1), we verified P3 methylation profile in the earlier available time point T1 respect to T1 of all other patients, finding 2138 DMGs (666 hyper- and 1473 hypo-methylated) (Supplementary Table S14b). Enrichment analysis of hypo-methylated genes included pathways related to cancer (Sagiv_CD24_targets_up) and progenitors of immune cells (Supplementary Figure S5b, Supplementary Table S16).

Subsequently, we intersected 5115 DMGs of P3 T4 vs all T4 with 2138 of P3 T1 vs all T1 and we found 1327 common DMGs (Fig. 6b, Supplementary Table S14). These genes were involved in both transcription and kinase activity, and in maintenance of cell number; notably, they showed an enrichment directly connected with cancer pathways such as lymphoma, MM, ovarian and breast cancer (Fig. 6b, Supplementary Table S17).

**Fig. 7:**
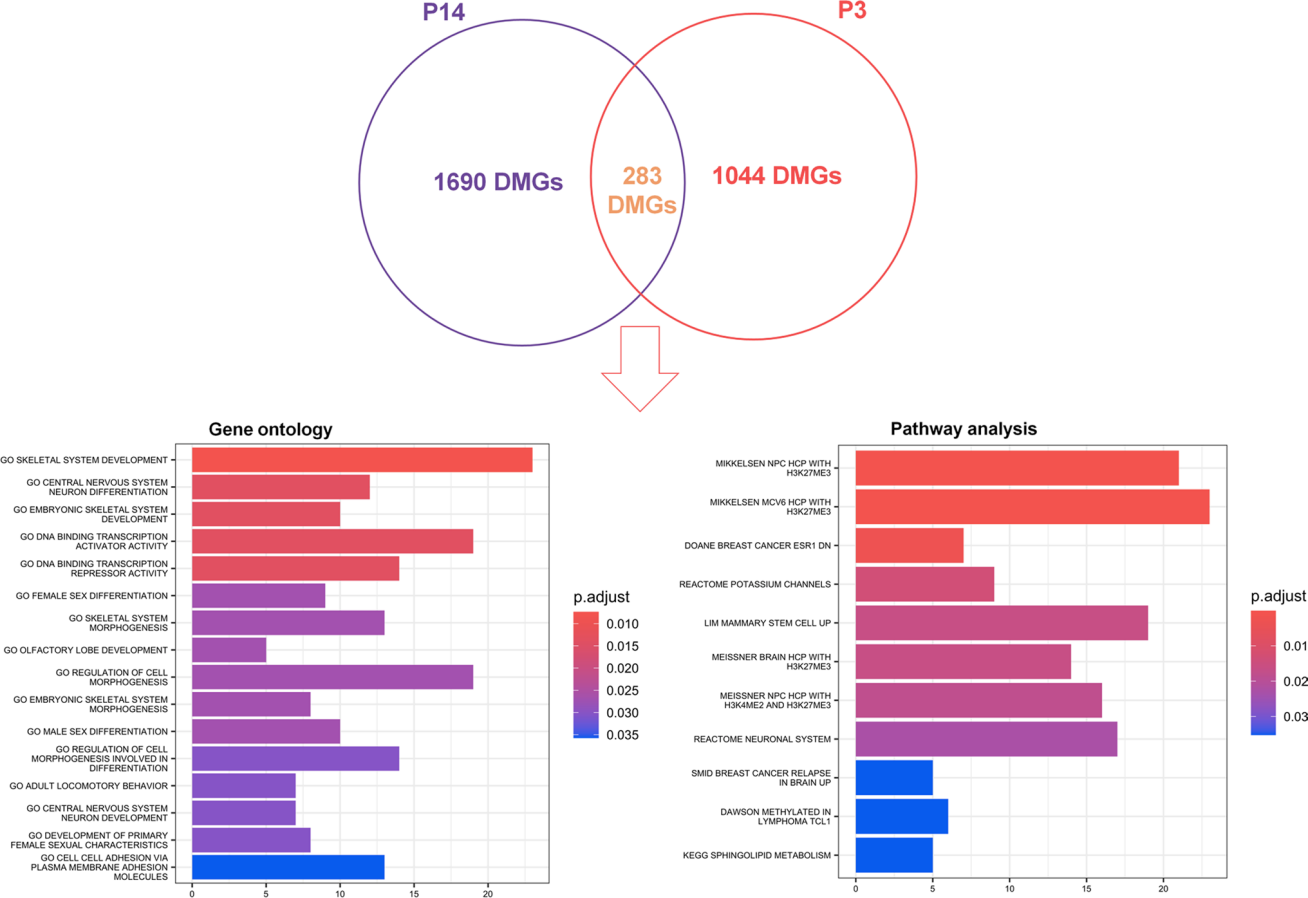
Gene signature of DMGs deriving from the intersection of common genes between the comparisons T4 and T1 of P14 and of P3. Venn diagram shows the number of common DMGs (n = 283) between the two comparisons and the non-overlapping numbers (n = 1690 and n = 1044) specifying the unique genes between T4 and T1 of P14 or of P3. GO and pathway analysis of hyper- and hypo-methylated DMGs (n = 283) from the intersection of common genes between T4 and T1 of P14 and of P3 are represented. The *y*-axis shows GO or pathways, and the *x*-axis represents the number of enriched genes. The color scale shows the significance of each GO or pathway signature showing the − log_10_ (adjusted *p*-value) with smaller *p*-value (red) representing more significant enrichment (Color figure online)

Finally, to understand whether there was a common methylation signature between these two patients, we crossed the 1973 DMGs of P14 with 1327 DMGs of P3 (Fig. 7). Interestingly, we found 283 common DMGs. Their functional enrichment showed genes involved in transcriptional activity and cell morphogenesis in GO, while in pathway analysis they participated in breast cancer and lymphoma (Fig. 7, Supplementary Table S18).

Collectively, follow–up of these two patients through the HSPC methylation analysis allowed us to highlight tumor pathways and GvHD genes.

## Discussion

AHSCT is an integral component of treatment of many hematological malignancies. To date, the understanding of biological mechanisms underlying stem cells holds fundamental relevance. The epigenetic landscape, including DNA methylation, regulates HSPC heterogeneity and hematopoietic cell fate decisions [28].

Here, the methylation dynamics of HSPCs pre- and post-transplant were explored to describe what occurs over time following transplant and to understand whether methylation changes could be associated with the biology of the transplant and with its possible failure. Our data showed that mPB-HSPCs of young donors (age ≤ 33 years) displayed a different level of global methylation respect to that of adult donor HSPCs (age ≥ 40 years), which was maintained in engrafted BM-HSPCs up to one year after AHSCT. Looking at the engrafted BM-HSPCs after AHSCT, a clear separation was evident between two groups likely associated to the donor age and to the nature of patient malignancy, together with patient conditioning regimen. Interestingly, in PCA of engrafted BM-HSPCs, post 30 days, this separation was even more evident.

It is well known that donor age and donor-recipient human leukocyte antigen match are of crucial importance for AHSCT outcome [29–31]. Recently, higher disease-free survival, better GvHD-free relapse free survival, and lower relapse rates were reported for patients with myelodysplastic syndrome, undergoing AHSCT with younger matched unrelated donors (MUD) compared with older matched sibling donors (MSD) [32]. These findings suggested that the choice of younger MUD should be considered in the donor selection algorithm for myelodysplastic syndrome, in which it is crucial to minimize relapse [29, 32]. Our setting included young and adult MUD (n = 4 age ≤ 33 years and n = 2 age ≥ 40 years, respectively) and one adult MSD (49 years, donor of P14). Our cohort of patients was too small for any definitive conclusion; however, we observed that donor age has no impact on stem cell harvest, product quality (data not shown), engraftment time (15–24–13 days for adult vs 14–17–14–11 days for young subjects) and overall survival. Except for two patients who died (P3 and P14, with donor age of 23 and 49 years, respectively), other patients were still alive.

Of note, recent literature shows that aging is associated with highly reproducible DNA methylation changes at specific sites of genome and allows the estimation of “biological aging” which may not match with chronological age. This latter is expected to be tightly linked to changes in major homeostatic mechanisms and, consequently, to be related to the chance of successful transplantation [33, 34].

Our results suggest that HSPCs from patients with myeloid malignancies, such as AML-P3–P7–P15, showed a similar methylation level among them, but different from patients with lymphoid malignancies, MM-P14, CLL-P8, HL-P5. It is well known that lymphoid and myeloid malignancies are different for pathobiology, clinical and relative BM microenvironment [35, 36]. HSPC methylation could be influenced by the microenvironment of the BM recipient and the applied conditioning regimen. The pre-transplant conditioning regimen should facilitate engraftment, reduce/eliminate tumor cells, and adequately suppress the recipient's immunity to prevent rejection of donor cells [2]. Specifically, the myeloablative regimen is intensive at the expense of mortality and morbidity, whereas reduced intensity conditioning (RIC) is safe but associated with a higher incidence of mixed chimerism and higher graft loss rates [37]. In our cohort, P5–8–14 were subjected to RIC, while P3–7–10–15 to myeloablative regimen. By grouping together all the samples of each specific time, a dominant global hypomethylation was observed among all groups, mainly in the promoter regions. By analyzing differences between recipient time points and donors, we observed that HSPCs from donors confirmed the strong hypo-methylation, with a slight difference among young and adults, while the other time points were characterized by a dominant hyper-methylation in the first 30 days after AHSCT that gradually decreased in the latter time points. A similar trend was reported in our previous paper, where methylation of donor BM-HSPCs and relative time points after AHSCT were analyzed [18]. Moreover, we analyzed methylation at promoter regions, converting the probes that mapped in these regions in genes to highlight eventually differences in gene expression.

Comparing the engrafted BM-HSPCs to the mPB-HSPCs, we immediately noted that 30 days post AHSCT, the engrafted BM-HSPCs underwent “methylation gene disturbance” resulting in 735 DMGs, most of which were hyper-methylated. This recipient HSPC methylation was particularly enriched in genes involved in cell adhesion, differentiation and different cytokine signaling including IL-2, IL-15, IL-7, IL-3, IL-5 and pathways, involved in hematopoietic homeostasis, growth, trafficking and immune function. IL-2 signaling is one of the most rapidly activated pathways that could allow an efficient cellular reaction in response to the microenvironmental changes in vivo; in addition, this system plays roles in lymphoid development, as well as in modulation of T-cell and NK-cell immune responses [38]. Our hypothesis is that methylome upset (735 DMGs) at 30 days post-transplant could be “a trail” of a greater disturbance that occurred before the 30th day as result of mPB-HSPC “adaptation” into the new BM recipient microenvironment. In fact, an interesting aspect is that, despite this methylation disturbance at 30 days, in the PB there was the normal hematopoietic recovery occurring in a range of 11–24 days in all patients (Table 1). This “gene methylation disturbance” persisted up to 180 days and was reduced at one year after AHSCT. This concept is also confirmed by the number of “stable genes” (as defined by us) which was about 180 after 6 months and decreased to 9 after one year. As previously reported, also donor BM derived-HSPCs showed a methylation trend similar to that of mPB-HSPCs: a methylation perturbation at 30 days after AHSCT but with a higher number of DMGs respect to that observed in mPB-HSPCs (1874 vs 735 DMGs) [18]. This perturbation was reduced after 60 days, reaching a similar level to that observed for mPB-HSPCs, and persisting up to 180 days [18]. It seems that mPB-HSPCs adapted to the patient’s BM “faster” than those from donors BM. Our hypothesis is that a first detachment of HSPCs from BM origin site, through mobilization, induced a first methylation change which made mPB-HSPCs more easily adaptable to the new BM than those of BM donors. This hypothesis may be consistent with faster clinical hematopoietic reconstitution obtained in patients which received mPB-HSPCs than BM derived-HSPCs. This last is one of several advantages of mPB-HSPC use over BM derived-HSPCs, including less invasive collection process for donor and a higher HSPC yield [39]. In the promoter region, our data indicate that most of the mPB-HSPC genes were in an active transcription phase, whereas, after 30 days, the BM-HSPC genes were dramatically silenced, subsequent time points showed instead a balance between gene activation and silencing.

Looking at common differential methylated genes in all single samples, we highlighted that mPB-HSPCs clustered all together sharing similar methylation with a dominance of hypo-methylated genes, with a slight difference between adult and young donors. Interestingly, BM-HSPCs collected 30 days post AHSCT associated to young donors and infused in patients which were subjected to myeloablative regimen (P3–10–15) shared more hyper-methylated genes (clustered very close) while the other HSPCs at 30 days from P5, P8 and P14 showed a different profile respect to previous cluster and they derived from adult donors and were infused in patients treated with RIC regimen. In particular, we believe that the separation of HSPCs at 30 days of P14 respect to the other ones could be due to the state of partial remission in which the patient was at the moment of AHSCT, and in which he persisted.

Noteworthy, global methylation analysis indicated that mPB-HSPCs have a “donor global methylation identity” which is maintained in the BM of patients while methylation of genes indicated that mPB-HSPCs, once in the BM of patients, are influenced by microenvironment and adapt to it. In particular, BM microenvironment of patients with same malignancy (see AML patients) and subjected to similar conditioning regimen (myeloablative) seems to act in a similar way on HSPC methylation status, while BM of patients with different malignancy (see HL-CLL-MM) and same conditioning regimen (reduced) induced, an early different HSPC methylation. This last difference decreases over time. In fact, at 180 days from AHSCT, the two clusters of HSPCs, one with P5 (HL)-8 (CLL)-14 (MM) and one with P3 (AML)-10 (ALL)-15 (AML) were very close.

We also deepened gene methylation modulation in patients to possibly correlate the observed methylation profile with outcome. In particular, we analyzed methylation of P14. Interestingly, P14 T4 showed 8726 DMGs in comparison with the same time point of other patients. These genes showed an enrichment directly connected with cancer such as lymphoma, prostate and gastric cancer. Looking at HSPCs post 30 days, surprisingly, we highlighted a tumor signature. Of note, a difference in global methylation between these HSPCs and others was already visible in the PCA analysis. Interestingly, common genes (1936 DMGs) between T4 and T1 time points increased their methylation levels (hyper- or hypo-methylation) during the interval from 30 to 163 days. We believe that this “gain of methylation” highlights an exacerbation of the process. These DMGs could be defined as potential “transplant methylation signature”. Of note, P14 persisted in partial remission after AHSCT, and the chimerism decreased passing from 96% (+ 30 days) to 93% (+ 163 days). A question arises: could the residual MM-plasma cells (PCs) and the microenvironment induce this HSPC methylation changes? Recently, we reported that MM-PCs communicate with HSPCs through the release of extracellular vesicles (EVs) [40], impairing HSPC differentiation. We have not investigated methylome of HSPCs after contact with PCs, but we cannot exclude that PCs, perhaps even through EVs, could compromise it, as this occurs in other settings [41]. In this direction, more in-depth studies are needed to clarify this issue.

Another interesting aspect of P14 is that in the immune enrichment of DMGs at 163 days, we found indications about TGF-β regulation. TGF-β is one of the most important cytokines regulating HSPC homeostasis, self-renewal [42] and recently, its expression was induced by latent HCMV infection of HSPCs and inhibited myelopoiesis [43]. Surprisingly, this patient died for reactivation of HCMV infection. Unfortunately, HCMV in AHSCT recipients remains a significant cause of morbidity and mortality with up to 80% of patients requiring antiviral therapy [44].

Furthermore, analyzing P3, which was affected by AML and in which all the AHSCT indicators, full chimerism and hematopoietic recovery, were good up to one year, in the last available sample of HSPCs we found a tumor pathway which, notably, was observed at 32 days yet. Several genes are enriched in different tumor phenotypes, like e.g., sarcoma, thyroid cancers and MLL gene. It is reported that MLL positivity is associated with a higher rate of relapse, lower leukemia-free survival and lower overall survival in leukemia patients after AHSCT [45]. In enrichment analysis of DMGs at 162 days, GvHD-associated genes (8 genes) were found. Surprisingly, the patient showed an acute GvHD in oral cavity.

Finally, intersecting specific DMGs of the two patients, we observed common tumor pathways involved in lymphoma and breast cancer including transcription factors and oncogenes (e.g., FOXD3/ESR1/HOXA9/BCL11B) [46]. We believe that these last genes could be a potential tumor methylation signature to look for and follow during AHSCT. Subsequent studies will be needed to confirm this hypothesis.

It should be noted that tumor pathways identified in the analysis of single patient, P14 or P3, were already found when we compared the BM-HSPCs (at 30 days post AHSCT) of all patients to PB-HSPCs of all donors. Therefore, methylation analysis highlighted the existence of tumor pathways despite their presence in only two out of seven patients analyzed, whose majority had a favorable outcome. This could mean that methylation analysis has a great “depth” and a high “specificity”.

Overall, using DNA methylation analysis, our results seem to be able to provide information about a recurrence of disease or a failure of transplant also in early phase, already at 30 days post AHSCT. Further analysis of a larger group of patients is needed to confirm our hypothesis.

## Conclusions

Methylation analysis could be a complementary tool to follow the progress of the transplant and potentially to predict, with a specific epigenetic signature, already at 30 days after the transplant, the risk of relapse. Such information could be very important especially in patients, who lack specific markers to track residual disease.

Although data are too limited to draw conclusive results, our study opens the perspective to investigate methylation profile as a new diagnostic indicator in the assessment of engraftment and prediction of graft failure in AHSCT and to ultimately understand how hematopoietic cell fate decisions are regulated during AHSCT.

### Supplementary Information

Supplementary figure and Table legends (Supplementary file1), Supplementary Figures S1–5 (Supplemantary file2), and Tables S1–18 (Supplementary file3-20) can be found in a separate file.

Below is the link to the electronic supplementary material.

Supplementary file1 (DOCX 18 KB) :  It is necessary to remove the yellow color from the sentences in this file

Supplementary file2 (PPTX 5613 KB)

Supplementary file3 (XLSX 3605 KB)

Supplementary file4 (XLSX 140 KB)

Supplementary file5 (XLSX 105 KB)

Supplementary file6 (XLSX 339 KB)

Supplementary file7 (XLSX 160 KB)

Supplementary file8 (XLSX 97 KB)

Supplementary file9 (XLSX 46 KB)

Supplementary file10 (XLSX 32 KB)

Supplementary file11 (PPT 164 KB)

Supplementary file12 (XLSX 1069 KB)

Supplementary file13 (XLSX 93 KB)

Supplementary file14 (XLSX 44 KB)

Supplementary file15 (XLSX 22 KB)

Supplementary file16 (XLSX 729 KB)

Supplementary file17 (XLSX 97 KB)

Supplementary file18 (XLSX 26 KB)

Supplementary file19 (XLSX 19 KB)

Supplementary file20 (XLSX 15 KB)

## Data Availability

The data that support the findings of this study are available from the corresponding author upon reasonable request.
