# Supplementary material for: DNA methylation of hematopoietic stem/progenitor cells from donor peripheral blood to patient bone marrow: implications for allogeneic hematopoietic stem cell transplantation
Source: Clin Exp Med. 2023 Apr 7;23(8):4493–510. doi: 10.1007/s10238-023-01053-w (PMC10725404; doi:10.1007/s10238-023-01053-w)
Supplement: Supplementary file 1 — Supplementary file1 (DOCX 18 KB) : It is necessary to remove the yellow color from the sentences in this file [file 10238_2023_1053_MOESM1_ESM.docx]

**Supplementary Figures**

**Supplementary Figure S1. (a-b)** Identification of confounding factors. Principal component analysis (PCA) performed on donor and patient HSPCs was constructed based on probes with largest standard deviation between samples to identify possible confounding factors. PCA plots reporting donor samples (T0) and recipients in **(a)** all time points (T1-T5) or **(b)** disease of patients. Each color corresponds **(a)** to a patient (P) which is indicated with its number or **(b)** to batch. AML: acute myeloid leukemia; ALL: acute lymphoblastic leukemia; CLL: chronic lymphocytic leukemia; MM: multiple myeloma; HL: Hodgkin lymphoma. **(c)** DNA methylation level (β-value) in both grouped donors (T0) and patients at each time point (T1, T2, T3, T4, T5). **(d)** DNA methylation level (β-value) of promoter (yellow) and gene body (orange) regions in grouped donors (T0) and patients at each time point (T1, T2, T3, T4, T5). The horizontal bar of **(c-d)** indicates the median level of methylation.

**Supplementary Figure S2. (a)** Methylation profile of donor and recipient HSPCs. Number of DMRs at each recipient time point vs donors (T1vsT0, T2vsT0, T3vsT0, T4vsT0 and T5vsT0) distinct in hypo- and hyper-methylated probes (dark and light blue, respectively). **(b)** Distribution of probes across CpG sites in both promoter and body regions. Distribution of hypo- and hyper-methylated probes across CpG sites (Island, Open Sea, Shelf and Shore) in promoter (yellow bars) and body regions (orange bars) in all time points compared to T0. In the vertical bar, the number of hypo-methylated and hyper-methylated probes is reported.

**Supplementary Figure S3. (a)** Functional analysis of hypo-DMGs obtained in all T1 with all T0. Pathway analysis and immunological signature of hypo-methylated genes obtained by comparison of all T1 with all T0. **(b)** Functional analysis of “stable” genes. Gene ontology (GO)**,** pathway analysis and immunological signature of hyper-methylated stable genes in T4.

The *y*-axis shows GO, pathway analysis or immunological signature, and the *x*-axis represents its enriched genes. The color scale showed the significance of each pathway or immunological signature showing the -log_10_ (adjusted *p*-value) with smaller *p*-value (red) representing more significant enrichment.

**Supplementary Figure S4.** Functional analysis of DMGs of P14. GO of **(a)** hyper- and **(b)** hypo-methylated obtained by comparison of P14 T4 vs all other patient T4. GO and pathway analysis of **(c)** hyper- and **(d)** hypo- methylated genes obtained by comparison of P14 T1 with all other T1.

The *y*-axis shows GO or pathway analysis and the *x*-axis represents its enriched genes. The color scale showed the significance of each GO or pathway analysis showing the -log_10_ (adjusted *p*-value) with smaller *p*-value (red) representing more significant enrichment.

**Supplementary Figure S5.** Functional analysis of DMGs of P3. **(a)** GO and immunological signature of hyper-methylated genes obtained by comparison of P3 T4 vs all other T4. **(b)** Pathway analysis and immunological signature of hypo- methylated genes obtained by comparison of P3 T1 vs all other T1.

The *y*-axis shows GO , pathways or immunological signature, and the *x*-axis represents its enriched genes. The color scale showed the significance of each pathway or immunological signature showing the -log_10_ (adjusted *p*-value) with smaller *p*-value (red) representing more significant enrichment.

**Supplementary Tables**

**Supplementary Table S1.** DMPs between all grouped T0 vs all grouped time points.

**Supplementary Table S2.** DMRs between all grouped T0 vs all grouped time points.

**Supplementary Table S3.** Median of β-value at different time points.

**Supplementary Table S4.** DMGs between all grouped T0 vs all grouped time points.

**Supplementary Table S5.** Median of β-value at different time points.

**Supplementary Table S6.** Functional analysis of DMGs in T1 vs T0.

**Supplementary Table S7.** Stable and revert DMGs.

**Supplementary Table S8.** Functional analysis of stable DMGs in T4.

**Supplementary Table S9.** Stable genes in T5.

**Supplementary Table S10.** DMGs of P14. P14 T4 vs all the other T4 **(A)**; P14 T1 vs all the other T1 **(B)**; intersection between A and B.

**Supplementary Table S11.** Functional analysis of DMGs (n=8726) obtained intersecting P14 T4 vs T4 of other patients.

**Supplementary Table S12.** Functional analysis of DMGs (n=2282) obtained intersecting P14 T1 vs T1 of other patients.

**Supplementary Table S13.** Functional analysis of 1973 DMGs obtained intersecting P14 T4 DMGs (n= 8726) vs P14 T1 DMGs (n=2282).

**Supplementary Table S14.** DMGs of P3. P3 T4 vs all the other T4 **(A)**; P3 T1 vs all the other T1 **(B)**; intersection between A and B.

**Supplementary Table S15.** Functional analysis of DMGs (n=5115) obtained intersecting P3 T4 vs T4 of other patients.

**Supplementary Table S16.** Functional analysis of DMGs (n=2138) obtained intersecting P3 T1 vs T1 of other patients.

**Supplementary Table S17.** Functional analysis of 1327 DMGs obtained intersecting P3 T4 vs all T4 DMGs (n= 5115) and those obtained by P3 T1 vs all T1 (n=2138).

**Supplementary Table S18.** Functional analysis of 283 common DMGs between 1973 DMGs of P14 and 1327 DMGs of P3.
