# Supplementary material for: DNA methylation of hematopoietic stem/progenitor cells from donor peripheral blood to patient bone marrow: implications for allogeneic hematopoietic stem cell transplantation
Source: Clin Exp Med. 2023 Apr 7;23(8):4493–510. doi: 10.1007/s10238-023-01053-w (PMC10725404; doi:10.1007/s10238-023-01053-w)
Supplement: Supplementary file 2 — Supplementary file2 (PPTX 5613 KB) [file 10238_2023_1053_MOESM2_ESM.pptx]

## Slide 1
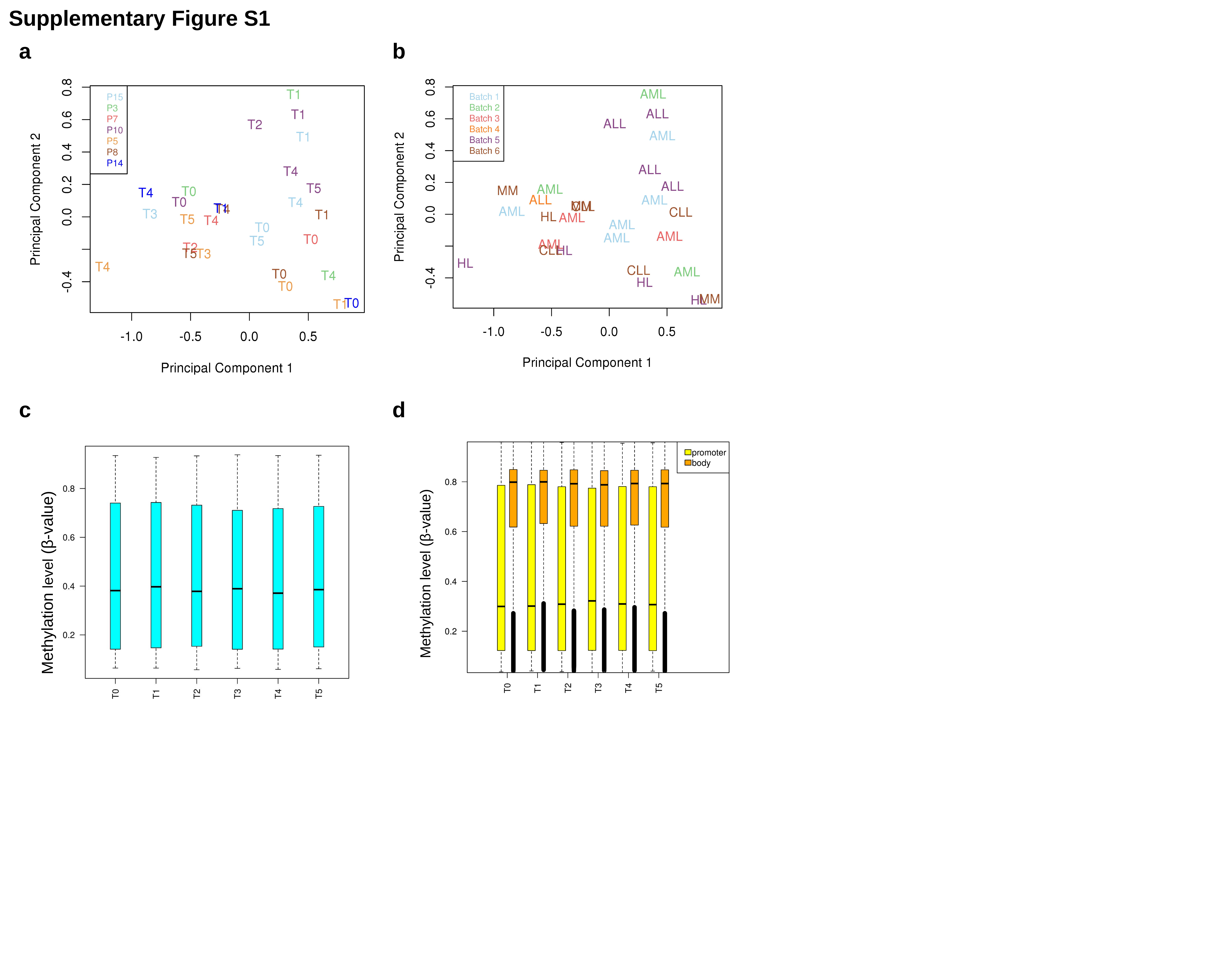

Supplementary Figure S1
a
b
c
d
Methylation level (β-value)
Methylation level (β-value)

## Slide 2
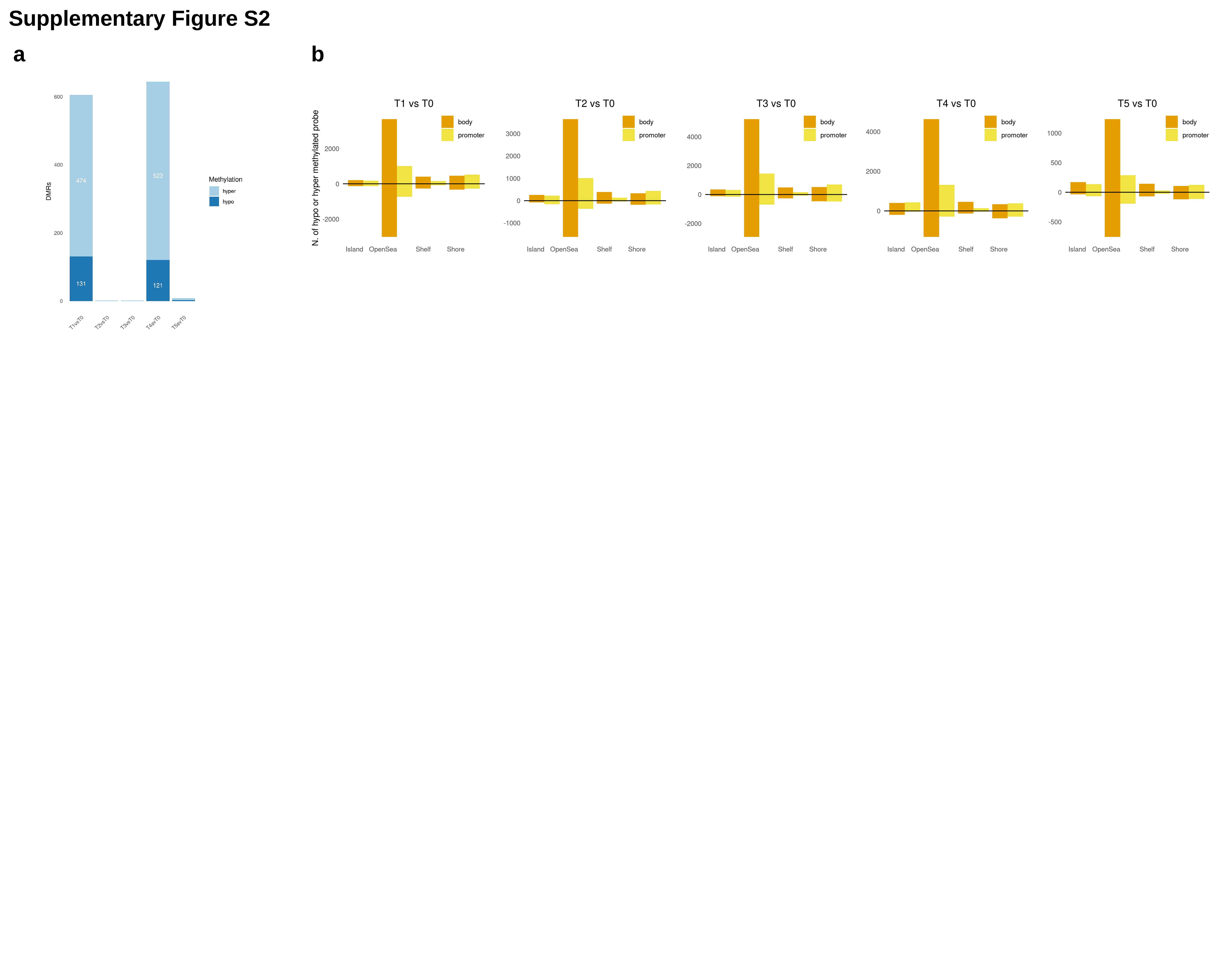

Supplementary Figure S2
a
b

## Slide 3
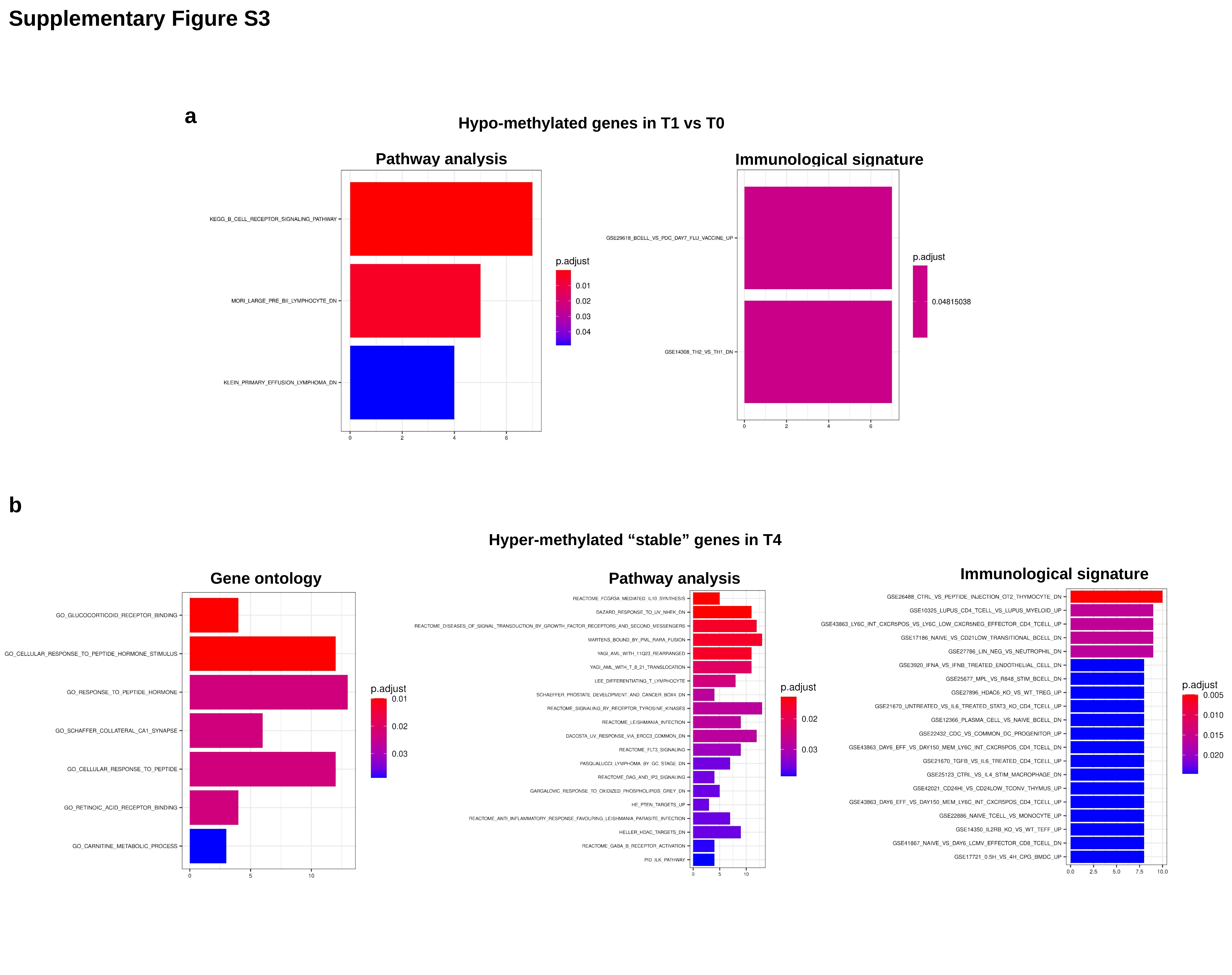

Supplementary Figure S3
a
Hypo-methylated genes in T1 vs T0
Pathway analysis
Immunological signature
b
Hyper-methylated “stable” genes in T4
Immunological signature
Gene ontology
Pathway analysis

## Slide 4
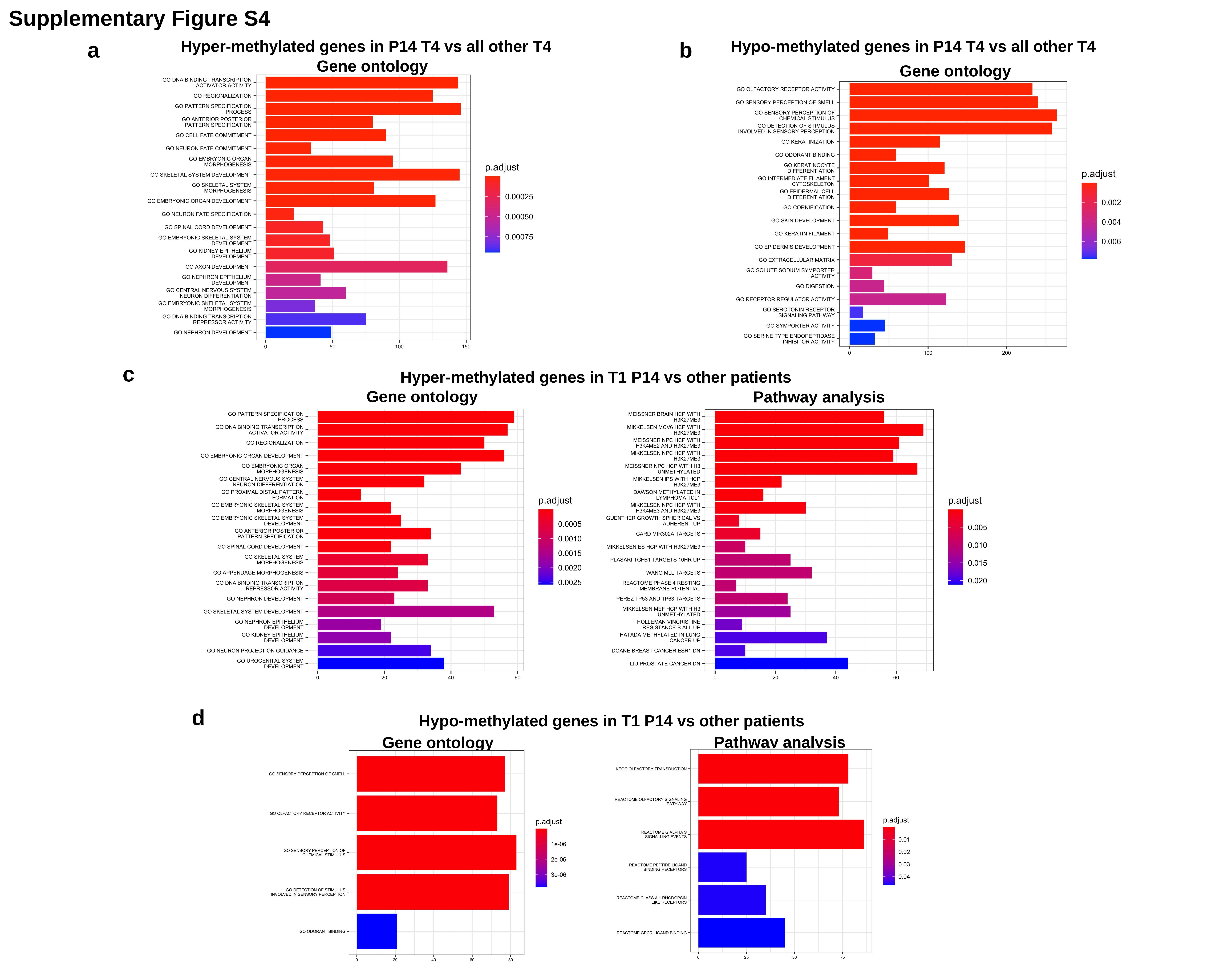

Supplementary Figure S4
a
b
Hyper-methylated genes in P14 T4 vs all other T4
Hypo-methylated genes in P14 T4 vs all other T4
Gene ontology
Gene ontology
c
Hyper-methylated genes in T1 P14 vs other patients
Gene ontology
Pathway analysis
d
Hypo-methylated genes in T1 P14 vs other patients
Pathway analysis
Gene ontology

## Slide 5
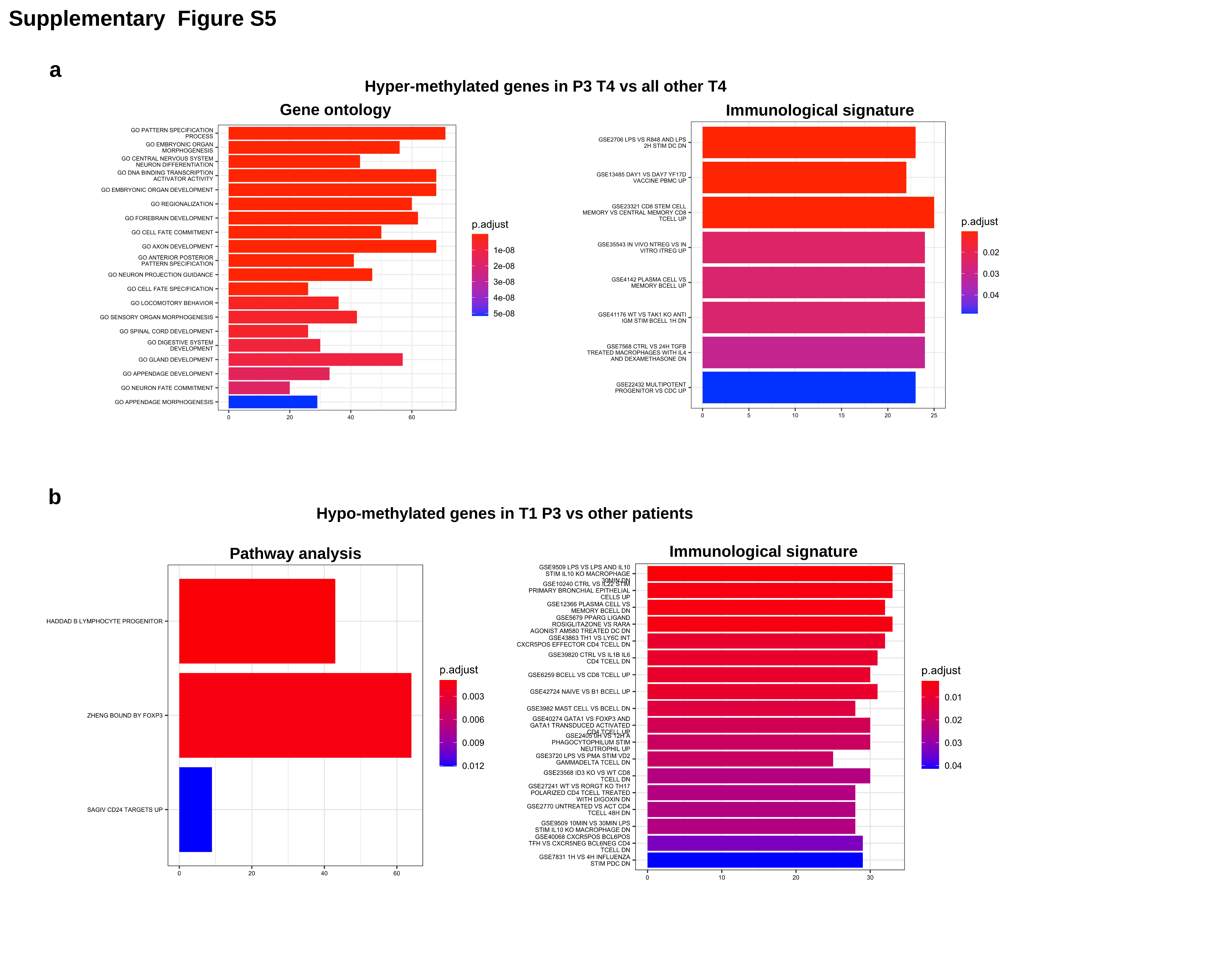

Supplementary Figure S5
a
Hyper-methylated genes in P3 T4 vs all other T4
Gene ontology
Immunological signature
b
Hypo-methylated genes in T1 P3 vs other patients
Immunological signature
Pathway analysis
