# Supplementary material for: DNA methylation of hematopoietic stem/progenitor cells from donor peripheral blood to patient bone marrow: implications for allogeneic hematopoietic stem cell transplantation
Source: Clin Exp Med. 2023 Apr 7;23(8):4493–510. doi: 10.1007/s10238-023-01053-w (PMC10725404; doi:10.1007/s10238-023-01053-w)
Supplement: Supplementary file 11 — Supplementary file11 (PPT 164 KB) [file 10238_2023_1053_MOESM11_ESM.ppt]

## Slide 1
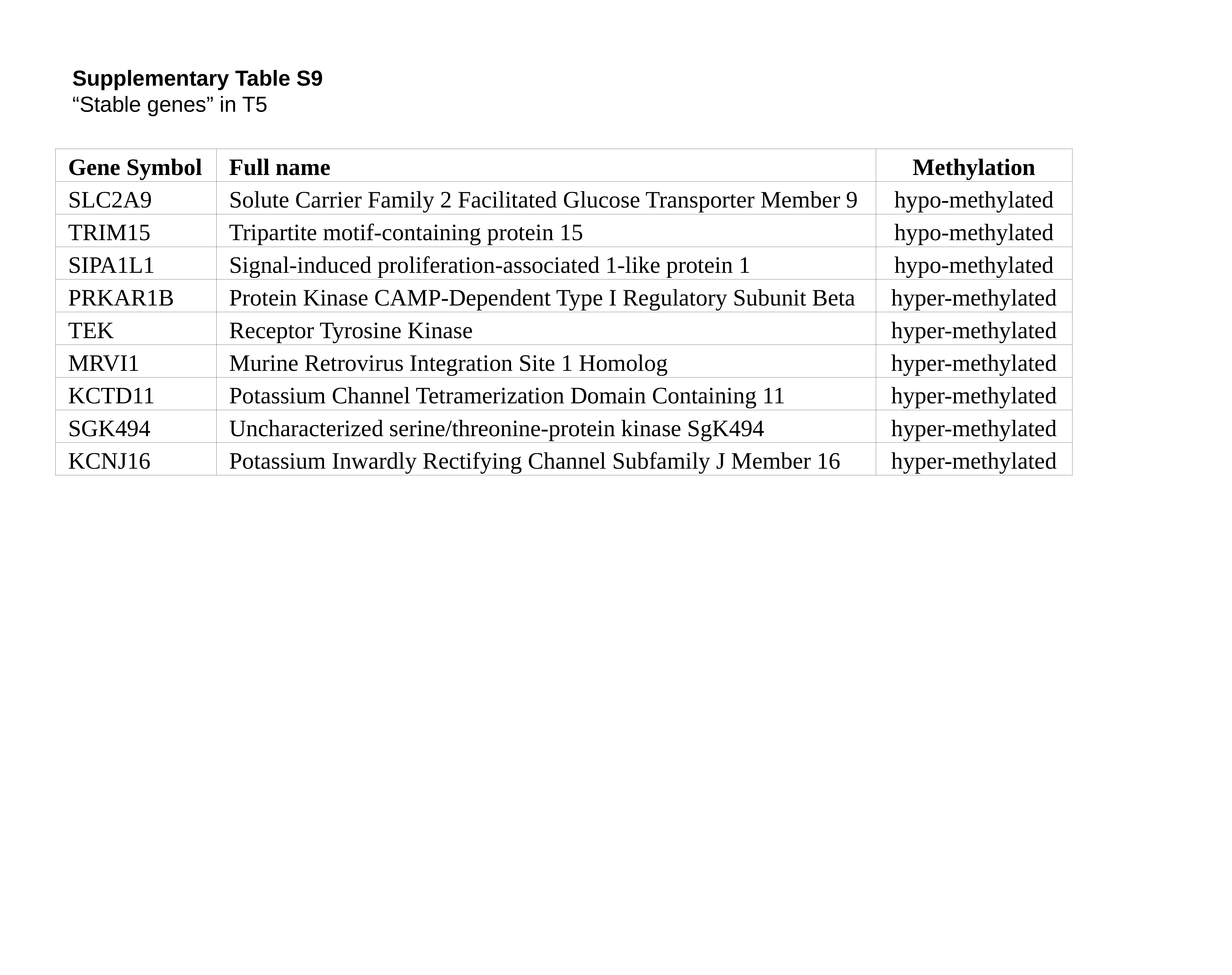

Supplementary Table S9
“Stable genes” in T5
| Gene Symbol | Full name | Methylation |
| --- | --- | --- |
| SLC2A9 | Solute Carrier Family 2 Facilitated Glucose Transporter Member 9 | hypo-methylated |
| TRIM15 | Tripartite motif-containing protein 15 | hypo-methylated |
| SIPA1L1 | Signal-induced proliferation-associated 1-like protein 1 | hypo-methylated |
| PRKAR1B | Protein Kinase CAMP-Dependent Type I Regulatory Subunit Beta | hyper-methylated |
| TEK | Receptor Tyrosine Kinase | hyper-methylated |
| MRVI1 | Murine Retrovirus Integration Site 1 Homolog | hyper-methylated |
| KCTD11 | Potassium Channel Tetramerization Domain Containing 11 | hyper-methylated |
| SGK494 | Uncharacterized serine/threonine-protein kinase SgK494 | hyper-methylated |
| KCNJ16 | Potassium Inwardly Rectifying Channel Subfamily J Member 16 | hyper-methylated |
